# Cell death upon epigenetic genome methylation: a novel function of methyl-specific deoxyribonucleases

**DOI:** 10.1186/gb-2008-9-11-r163

**Published:** 2008-11-21

**Authors:** Eri Fukuda, Katarzyna H Kaminska, Janusz M Bujnicki, Ichizo Kobayashi

**Affiliations:** 1 Laboratory of Social Genome Sciences, Department of Medical Genome Sciences, University of Tokyo, 4-6-1 Shirokanedai, Minato-ku, Tokyo, 108-8639, Japan; 2 Graduate Program in Biophysics and Biochemistry, Graduate School of Science, University of Tokyo, 4-6-1 Shirokanedai, Minato-ku, Tokyo, 108-8639, Japan; 3 International Institute of Molecular and Cell Biology, Trodena 4, 02-109 Warsaw, Poland; 4 Faculty of Biology, Adam Mickiewicz University, Umultowska 89, 61-614 Poznan, Poland; 5 Institute of Medical Science, University of Tokyo, 4-6-1 Shirokanedai, Minato-ku, Tokyo, 108-8639, Japan

## Abstract

**Background:**

Alteration in epigenetic methylation can affect gene expression and other processes. In Prokaryota, DNA methyltransferase genes frequently move between genomes and present a potential threat. A methyl-specific deoxyribonuclease, McrBC, of *Escherichia coli *cuts invading methylated DNAs. Here we examined whether McrBC competes with genome methylation systems through host killing by chromosome cleavage.

**Results:**

McrBC inhibited the establishment of a plasmid carrying a PvuII methyltransferase gene but lacking its recognition sites, likely through the lethal cleavage of chromosomes that became methylated. Indeed, its phage-mediated transfer caused McrBC-dependent chromosome cleavage. Its induction led to cell death accompanied by chromosome methylation, cleavage and degradation. RecA/RecBCD functions affect chromosome processing and, together with the SOS response, reduce lethality. Our evolutionary/genomic analyses of McrBC homologs revealed: a wide distribution in Prokaryota; frequent distant horizontal transfer and linkage with mobility-related genes; and diversification in the DNA binding domain. In these features, McrBCs resemble type II restriction-modification systems, which behave as selfish mobile elements, maintaining their frequency by host killing. McrBCs are frequently found linked with a methyltransferase homolog, which suggests a functional association.

**Conclusions:**

Our experiments indicate McrBC can respond to genome methylation systems by host killing. Combined with our evolutionary/genomic analyses, they support our hypothesis that McrBCs have evolved as mobile elements competing with specific genome methylation systems through host killing. To our knowledge, this represents the first report of a defense system against epigenetic systems through cell death.

## Abstract 2

The McrBC methyl-specific deoxyribonuclease from *Escherichia coli* can respond to genome methylation by host killing.

## Background

Recent studies have revealed that epigenetic genome methylation is associated with many aspects of life processes through effects on gene expression and other steps [1-3]. Especially, epigenetic methylation is involved in silencing of selfish genetic elements and other aspects of intragenomic conflicts. Experimental alteration of epigenetic DNA methylation systems can cause a wide variety of changes [4-8]; for example, in Prokaryota, DNA methyltransferase action can change the transcriptome [7]. Horizontal gene transfer contributes considerably to the building up of prokaryotic genomes [9,10]. In particular, the DNA methyltransferase genes frequently move between genomes [11-15] and could, therefore, present potential threats to prokaryotic genomes, although they can also be beneficial to bacteria in many ways, including in cell cycle regulation and cell differentiation [3,8].

**Figure 1:**
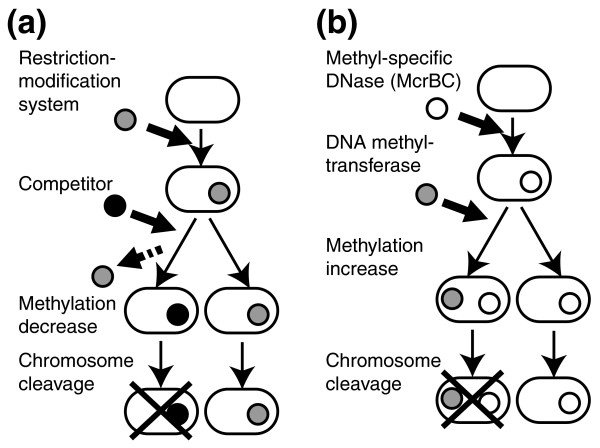
Host killing by RM systems and by methyl-specific DNases (McrBC) in competition. **(a) **When a resident RM gene complex is replaced by a competitor genetic element, a decrease in the modification enzyme level results in exposure of newly replicated chromosomal restriction sites to lethal cleavage by the remaining restriction enzyme molecules. The intact genome copies will survive in uninfected neighboring clonal cells. **(b) **When a DNA methylation system enters a cell and begins to methylate chromosomal recognition sites, McrBC senses the change and triggers cell death by chromosomal cleavage. The intact genome copies will survive in uninfected neighboring clonal cells.

Prokaryotic DNA methyltransferases often form a restriction-modification (RM) system together with a restriction enzyme [16,17]. Some RM systems behave as mobile elements, as suggested by their amplification, mobility, and involvement in genome rearrangements, as well as their mutual competition and regulation of gene expression [13-15,18-21]. Some type II RM systems cleave chromosomes of their host cells when their genes are eliminated by a competitor genetic element [20,22,23], as illustrated in Figure 1a. Such host killing, called 'post-segregational killing' or 'genetic addiction', has been recognized to be involved in stable maintenance in many plasmids [24]. The RM systems have evolved regulatory systems to suppress their potential to kill the host. When they enter a new host, they prevent host cell killing by expressing their methyltransferase first and delaying expression of their restriction enzyme [19,25-27].

Host chromosome cleavage by RM systems is not trivial. In general, cleavage of chromosomes by cellular DNases is prevented in various ways: inhibitor binding, compartmentalization, proteolysis, DNA modification and DNA structure specificity. Indeed, host killing by RM systems after loss of their genes is not always obvious because hosts have apparently adapted to counteract it in various ways. Recombination repair of chromosomal breakage can reduce the lethal effects of chromosome cleavage [28]. Host killing by an RM gene complex is suppressed by a solitary methyltransferase recognizing the same sequence [29,30]. Proteolytic digestion of restriction enzymes suppresses chromosome cleavage by *Eco*KI, a type I RM system, even in the absence of the cognate methyltransferase [31]. These host defense systems against RM systems cannot, however, avoid host genome methylation and its potentially deleterious effects.

In the present work, we provide evidence for the existence of a group of genetic elements that compete with epigenetic DNA methylation systems (for example, with DNA methyltransferases from RM systems) through host cell killing. These anti-methylation elements are methyl-specific endodeoxyribonuclease McrBC of *Escherichia coli *[32] and its homologs. McrBC cleaves DNA between two separate R^m^C (R = A or G, ^m^C = ^m4^C or ^m5^C) sites *in vitro *[33], which are modified by many DNA methyltransferases from different RM systems [16,17]. This activity was first recognized for restriction of incoming bacteriophage genomes carrying hydroxymethylcytosine instead of cytosine [34,35]. McrBC may also protect cells against infection by methylated DNA elements, such as viral genomes and plasmids, through such direct cleavage. However, such methylated DNAs are not usually strongly restricted by McrBC [36,37]; therefore, we hypothesized that McrBC may mediate suicidal defense in response to epigenetic genome methylation systems, such as RM systems, as illustrated in Figure 1b. When such a system enters the cell and begins to methylate the host genome, McrBC would sense these epigenetic changes and trigger cell death through chromosomal cleavage. Intact (unmethylated) genomes with *mcrBC *genes would survive in the neighboring clonal cells.

Defense against invasion of genetic elements through cell death, as illustrated in Figure 1a,b, has been reported for multicellular eukaryotic cells, such as virus-infected mammalian cells and plant cells [38]. Similar phenomena against virus infection have been known for bacteria under the name of 'phage exclusion' or 'phage abortion' [39]. Bacteriophage reproduction is aborted by the action of a cell death gene. As a result, this gene would survive within the clonal cells that would, otherwise, all die by secondary infection. For example, the *prr *gene in some *Escherichia coli *strains senses bacteriophage T4 infection and triggers cell death by cleaving host tRNA^Lys ^[40].

We first asked whether McrBC-mediated cell death through cleavage of methylated chromosomes takes place upon entry/induction of a methyltransferase gene and aborts its establishment/activation. After obtaining positive experimental results, we asked how important this role has been in the spread and maintenance of McrBC genes. Our analyses of their molecular evolution and genomic contexts support the hypothesis that, during evolution, they have behaved as mobile elements. Taken together, these results support our hypothesis that McrBCs have evolved as mobile elements that compete with specific genome methylation systems through host killing.

## Results

In the first half of the Results section, we address the first question of whether McrBC-mediated cell death through cleavage of methylated chromosomes takes place upon entry/induction of an epigenetic methyltransferase gene and causes this gene's establishment/activation to be aborted.

### McrBC-mediated inhibition of establishment of a DNA methyltransferase gene

We first asked about the biological consequences of McrBC, that is, whether or not establishment of a transferred methyltransferase gene is aborted through the action of McrBC. As the methyltransferase, we chose PvuII methyltransferase (M.PvuII) of the PvuII RM system. It recognizes CAGCTG and generates CAG^m4^CTG [37,41], a target sequence of McrBC [33].

Several reports have indicated that phages or plasmids carrying a DNA methyltransferase gene could not be propagated in an *mcrBC*^+ ^strain of *E. coli *[42]. Whether the block to propagation is due to repeated methylation of the introduced DNA and subsequent cleavage [42] or due to host genome methylation and cleavage, as we have hypothesized in this work, has not been addressed.

**Figure 2:**
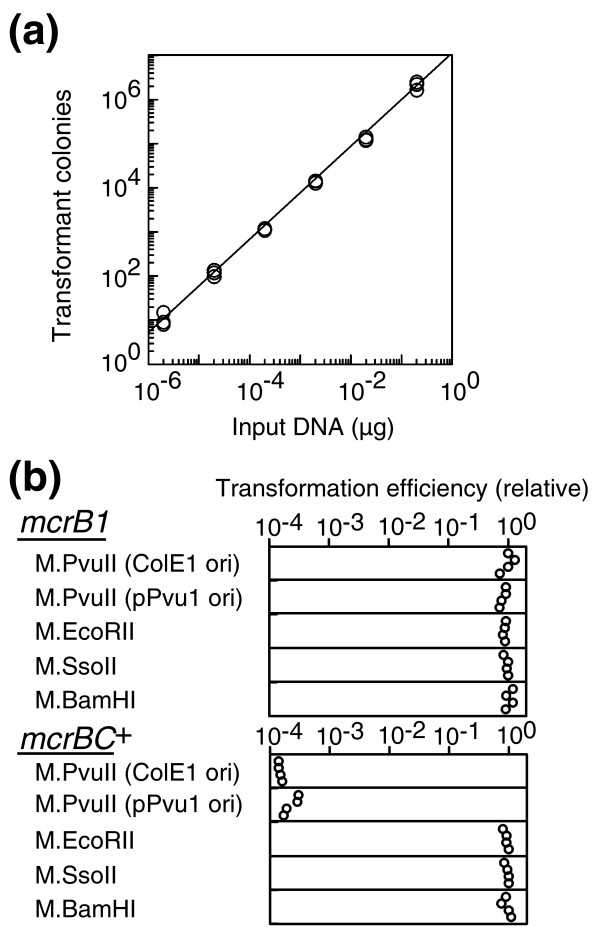
McrBC-mediated blocking of establishment of an epigenetic genome methylation system. **(a) **Quantitative transformation. Varying amounts of pUC19 (2 pg, 20 pg, 200 pg, 2 ng, 20 ng, and 200 ng) were used to transform *E. coli *DH5α by electroporation. Experiments were conducted in triplicate. **(b) **Transformation of plasmids carrying the PvuII methyltransferase gene. Plasmids (100 ng) carrying one of several modification methyltransferase genes were used to transform *E. coli *ER1562 (*mcrB1*) and ER1563 (*mcrBC*^+^). The relative transformation efficiency was calculated as the ratio of the transformation efficiency of the test plasmid to that of the empty vector. M.PvuII (ColE1) indicates pEF43, while M.PvuII (pPvu1) indicates pEF65 (Table 1). The empty vector for the latter is pEF67, while that for the former is pEF33. The vector for the remaining plasmids is pBR322. The measurements from two separate experiments conducted in duplicate are shown. All (20/20) of the rare transformants of *mcrBC*^+ ^by pEF43 examined were found to have lost McrBC activity.

We introduced a plasmid carrying the PvuII methyltransferase (M. PvuII, CAG^m4^CTG) gene but lacking PvuII recognition sites (pEF43 in Table 1) in a quantitative transformation assay (Figure 2a). The transformation efficiency decreased by four orders of magnitude in an *mcrBC*-dependent manner (Figure 2b). The decrease did not occur in the case of genes for three other cytosine methyltransferases, M.EcoRII (C^m5^CWGG), M.SsoII (C^m5^CNGG), and M.BamHI (GGAT^m4^CC), consistent with the sequence specificity of McrBC [33]. We found that a plasmid carrying a PvuII methyltransferase gene and two PvuII recognition sites was also inhibited in its establishment by the same order of magnitude (date not shown). Our results indicate that methylated sites on the transferred DNA were not required for the McrBC-dependent inhibition of its establishment and propagation. These results demonstrate that McrBC can abort establishment of the transferred element with the methyltransferase gene and, furthermore, suggest that this is through McrBC-mediated cleavage of methylated chromosomal DNA, as opposed to that on the transferred DNA.

**Table 1:** Plasmids

Plasmids	Prototype	Relevant characteristics	Drug resistance	Source, reference
pBR322	pBR322		Ap, Tc	Laboratory collection [107]
pUC19	pUC19		Ap	Laboratory collection [108]
PACYC184	pACYC184		Cm, Tc	Laboratory collection [109]
pSC101	pSC101		Tc	National Institute of Genetics [110]
pBAD18	pBR322	P_BAD_	Ap	National Institute of Genetics [51]
pIK8004	pBR322	NotI linker (GCGGCCGC) in DraI site	Ap	M. Kawai (our laboratory)
PYNEC302	pUC19	*pvuIIR*^-^*MC*	Ap	Y Nakayama [19]
PYNEC313	pBR322	*pvuIIRMC*	Ap	Y Nakayama [19]
PYNEC404	pUC19	*bamHIR*^-^*MC*	Ap	Y Nakayama [19]
pNY43	pBR322	*ecoRIIR*^-^*M*	Ap	Y Naito [111]
pNY44	pBR322	*ssoIIR*^-^*M*	Ap	Y Naito [111]
pEF1	pBR322	P_BAD_, *pvuIIM*	Ap	This work
pEF23	pBR322	P_BAD_, *pvuIIM*	Ap	This work
pEF24	pSC101	P_BAD_, *pvuIIM*	Ap	This work
pEF30	pBR322	*bamHIR*^-^*MC*	Ap	This work
pEF33	pBR322	No PvuII site	Ap, Tc	This work
pEF43	pBR322	*pvuIIR*^-^*MC*, no PvuII site	Ap	This work
pKD13		*OriRγ*	Ap, Km	*E. coli *Genetic Stock Center [90]
pKD46		pSC101(Ts) ori, *araC*-P_BAD_-*redαβ*	Ap	*E. coli *Genetic Stock Center [90]
pCP20		pSC101(Ts) ori, P_r_-FLP	Ap	*E. coli *Genetic Stock Center [112]
pBAD30	pACYC184	P_BAD_	Cm	National Institute of genetics [51]
pSI4	pUC19	*sinIRM*	Ap	C. Karreman [113]
pNW106RM2-3	pBR322	*mspIRM*	Ap	New England Biolabs [114]
pEF46		P_BAD_-*mcrBC*	Cm	This work
pUC4K	pBR322		Ap, Km	Laboratory collection [115]
pEF60	pBR322		Km	This work
pPvuCat16	pPvu1	pPvu1 ori, *pvuIIM*	Cm	Robert Blumenthal [43]
pPvuCat17	pPvu1	pPvu1 ori	Cm	Robert Blumenthal [43]
pEF65	pPvu1	pPvu1 ori, *pvuIIM*	Km	This work
pEF67	pPvu1	pPvu1 ori	Km	This work

Ap, ampicillin-resistance; Cm, chloramphenicol-resistance; Km, kanamycin-resistance; Tc, tetracycline-resistance; Ts, temperature-sensitive.

The PvuII RM gene complex was found on pPvu1, a low-copy plasmid from Proteus vulgaris [37] that can also replicate in E. coli [43]. Proteus vulgaris and E. coli both belong to the Enterobacteriaceae family and also share an ecological niche, the intestine of humans and related animals. Therefore, these experiments are intended to reproduce events that are likely to take place in the natural environment, although they involved the use of multicopy (ColE1-derived) plasmids. Transformation of a pPvu1 derivative plasmid carrying M.PvuII and a drug-resistance gene as a selective marker and lacking PvuII sites (pEF65 in Table 1) was blocked by McrBC as strongly as the above multi-copy plasmid (Figure 2b). This suggests that the strong inhibition is biologically relevant.

### McrBC-mediated chromosome cleavage after phage-mediated transfer of the DNA methyltransferase gene

The above inhibition of establishment of the methyltransferase gene is likely caused by lethal cleavage of chromosomes that become methylated. Next, we asked whether McrBC indeed cleaves host chromosomes in order to abort the propagation of a transfered epigenetic genome methylation gene. In order to examine this issue, we introduced the M.PvuII gene into *E. coli *by a λ phage vector.

**Figure 3:**
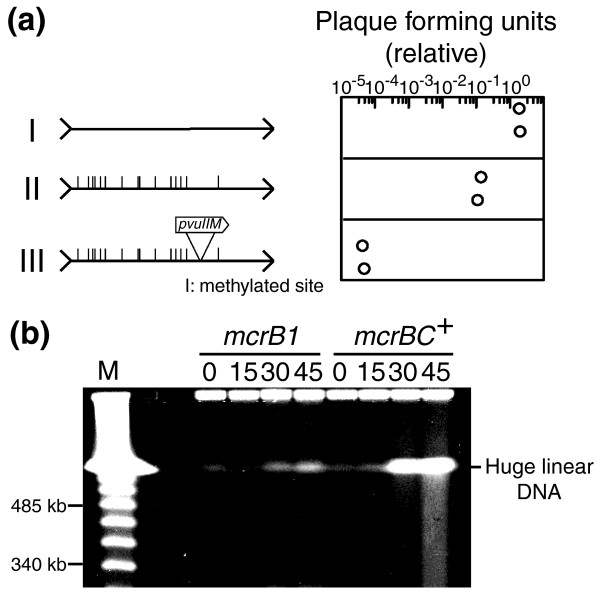
McrBC-mediated inhibition of phage growth and chromosome cleavage. **(a) **Phage λ titer on ER1563 (*mcrBC*^+^) divided by its titer on ER1562 (*mcrB1*) is plotted for two independent experiments. (I) A λ strain with 15 PvuII sites (LIK891; see Materials and methods); (II) the same λ strain but modified by PvuII methyltransferase; (III) the same λ strain with insertion of PvuII methyltransferase gene (LEF1). **(b) **Chromosome degradation in ER1562 (*mcrB1*) and ER1563 (*mcrBC*^+^). 5 × 10^8 ^cells were infected with LEF1 at a multiplicity of infection of 5. At the indicated time intervals (in minutes) after infection of phage carrying the PvuII methyltransferase gene (LEF1), chromosomal DNA was prepared and subjected to pulsed-field agarose gel electrophoresis. M, λ DNA ladder.

We first prepared the λ phage strain LIK891 with 15 PvuII sites (Materials and methods) in a host carrying PvuII methyltransferase (Materials and methods). Its modification status was confirmed by its resistance to PvuII restriction both *in vitro *and *in vivo *as follows. When the phage genome DNA prepared from the purified λ preparation was reacted with PvuII, no change was observed in its gel electrophoresis pattern under a condition where unmodified phage genome DNA was completely cleaved. The PvuII-modified phage preparation did not show detectable decreases in plaque formation efficiency in a host carrying the PvuII RM system. In an *E. coli mcrBC*^+ ^strain, the PvuII-modified λ phage preparation showed only a 10-fold decrease in plaque formation efficiency (Figure 3a). Consistent with previous reports [36,37], this observation indicates that McrBC cannot efficiently restrict a methylated phage genome.

However, λ phage strain LEF1, which carries the PvuII methyltransferase gene, was restricted 10,000-fold (Figure 3a). This result agrees with earlier reports indicating that phages carrying a DNA methyltransferase gene could not be propagated in an *mcrBC*^+ ^strain of *E. coli *[43]. As we noted in the previous section, whether the block to propagation is due to repeated methylation of the introduced DNA and subsequent McrBC-mediated cleavage [43] or due to host genome methylation and its McrBC-mediated lethal cleavage has not been addressed.

When we examined chromosomes of the infected cells by pulsed-field gel electrophoresis, we observed accumulation of huge linear DNA corresponding to broken chromosomes (indicated in Figure 3b in the lanes at 30 and 45 minutes after infection) and of smaller DNAs of variable size (smear in Figure 3b in the lane at 45 minutes after infection), which likely reflect chromosome degradation. Their appearance was *mcrBC*^+^-dependent (*mcrB1 *lanes in Figure 3b). This observation strongly suggests that M.PvuII-mediated chromosome methylation triggered chromosome cleavage by McrBC, which was followed by chromosome degradation. This, in turn, indicates that the inhibition of their multiplication (Figure 3a) is caused by host death.

Parenthetically, we noticed a band deriving from both the *mcrB*^- ^and *mcrBC*^+ ^strains in the middle of the same gel and another species at the lowest position from the *mcrBC*^+ ^cells (data not shown). From their mobility, we inferred that these bands represent the excised circular form and the cleaved linear form of e14, a defective lambdoid phage [44,45]. Because e14 has one PvuII site, its linear form is expected to appear after McrBC-mediated cleavage [46]. Because the lambdoid phages have similar gene organization [47-49] and regulation [50], it would not be very surprising if gene expression from the incoming λ somehow led to the expression of the excision function of e14.

### McrBC-mediated cell death and chromosome degradation following induction of the DNA methyltransferase

The above two sets of experiments strongly suggested that McrBC mediates inhibition of propagation of the PvuII DNA methyltransferase gene through lethal cleavage of methylated chromosomes. We next asked whether induction of the PvuII methyltransferase leads to chromosome methylation followed by its McrBC-mediated cleavage and cell death. Furthermore, we asked whether we could find a close correlation between these three processes: methylation, cleavage and death.

**Figure 4:**
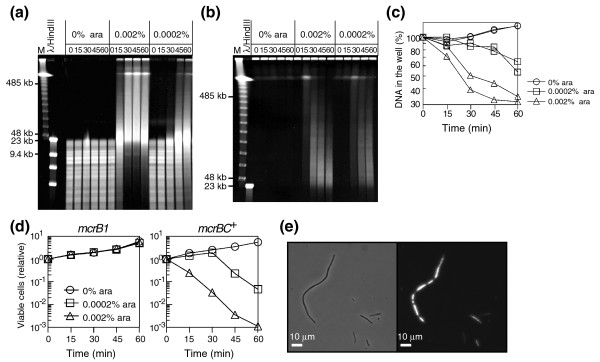
Expression of PvuII methyltransferase causes chromosome methylation and *mcrBC*-dependent chromosome breakage and cell death. **(a) **Confirmation of chromosome methylation. BIK18260 (*mcrB1*) cells carrying pEF24 (*pvuIIM *under the pBAD promoter; see Table 1), were grown in LB broth under antibiotic selection to the mid-exponential phase, diluted to OD600 = 0.1, and further grown in the presence of 0.002% or 0.002% arabinose (ara) to induce expression of M.PvuII. At the indicated time intervals (in minutes), chromosomal DNA was prepared, digested with PvuII endonuclease (TaKaRa Bio), and subjected to pulsed-field agarose gel electrophoresis. M, λ DNA ladder. **(b) **Chromosome DNA in BIK18261 (*mcrBC*^+^) carrying pEF24 after induction of PvuII methyltransferase. **(c) **Ethidium-bromide fluorescence in the well was measured for the experiments in (b). **(d) **Loss of cell viability. The number of viable cells was monitored in duplicate in two independent experiments. Each value was divided by the value at time zero. **(e) **Cell shape. The cells were recovered 60 minutes after addition of a higher (0.002%) concentration of arabinose. They were stained with DAPI to visualize nucleoids and were observed by phase-contrast (left) and fluorescence (right) microscopy. The scale bar indicates 10 μm.

First, we cloned the *pvuIIM *gene downstream of the arabinose-inducible BAD promoter [51]. We prepared host strains for this experiment based on the work of Khlebnikov *et al. *[52]. These authors succeeded in achieving homogeneous expression from the BAD promoter and obtained a linear increase in the expression level in response to arabinose concentration by deleting *araBAD *and *araFGH *operons and substituting the *araE *promoter with a constitutive promoter [52]. We introduced these mutations to construct isogenic *mcrBC*^+/- ^strains (BIK18260 and BIK18261 in Table 2). At three concentrations of arabinose (0%, 0.0002%, and 0.002%) we were able to demonstrate correlation between genome methylation, genome breakage and cell death (Figure 4) as detailed below.

**Table 2:** Bacteria

*E. coli *strains	Genotype	Source and/or reference
ER1562	F^- ^λ^-^*endA1 thi-1 supE44 hsdR2 mcrB1 mcrA1272*::Tn10	New England Biolabs [89]
ER1563	F^- ^λ^-^*endA1 thi-1 supE44 hsdR2 mcrA1272*::Tn10	New England Biolabs [89]
BIK18046	ER1562 but Tc^s^	Tc^s ^with fusaric acid
BIK18051	ER1563 but Tc^s^	Tc^s ^with fusaric acid
BIK18116	ER1562 Δ(*recB-recC*)::*kan*	Km^R ^with pKD46-mediated transformation with PCR product from deletion allele primers and pKD13 template
BIK18118	ER1563 Δ(*recB-recC*)::*kan*	Km^R ^with pKD46-mediated transformation with PCR product from deletion allele primers and pKD13 template
BIK18120	ER1562 Δ*recA*::*kan*	Km^R ^with pKD46-mediated transformation with PCR product from deletion allele primers and pKD13 template
BIK18125	ER1563 Δ*recA*::*kan*	Km^R ^with pKD46-mediated transformation with PCR product from deletion allele primers and pKD13 template
BIK18142	ER1562 Δ*araBAD*::*kan*	Km^R ^with pKD46-mediated transformation with PCR product from deletion allele primers and pKD13 template
BW27269	*lacI*^q^*rrnB3 *Δ*lacZ4787 hsdR514 *Δ*(araBAD)567*	*E. coli *Genetic Stock Center [52]
	Δ(rhaBAD)568Δ*(araFGH)*::*kan903*	
BW27535	*lacI*^q^*rrnB3 ΔlacZ4787 hsdR514 Δ(araBAD)567*	*E. coli *Genetic Stock Center [52]
	Δ(rhaBAD) 568 g(Δ*araEp kan *P_cp13_-*araE*)	
BIK18244	BIK18046 Δ*araBAD*::*kan*	P1 from BIK18116 to ER1562
BIK18246	BIK18051 Δ*araBAD*::*kan*	P1 from BIK18116 to ER1563
BIK18248	BIK18046 Δ*araBAD*	BIK18244 Km^s ^with pCP20
BIK18249	BIK18051 Δ*araBAD*	BIK18246 Km^s ^with pCP20
BIK18250	BIK18046 Δ*araBAD *ϕ(Δ*araEp kan *P_cp13_-*araE*)	P1 from BW27535 to BIK18248
BIK18252	BIK18051 Δ*araBAD *ϕ(Δ*araEp kan *P_cp13_-*araE*)	P1 from BW27535 to BIK18249
BIK18254	BIK18046 Δ*araBAD *ϕ(Δ*araEp *P_cp13_-*araE*)	BIK18250 Km^s ^with pCP20
BIK18255	BIK18051 Δ*araBAD *ϕ(Δ*araEp *P_cp13_-*araE*)	BIK18252 Km^s ^with pCP20
BIK18256	BIK18046 Δ*araBAD *ϕ(Δ*araEp *P_cp13_-*araE*) Δ*(araFGH)*::*kan903*	P1 from BW27269 to BIK18254
BIK18258	BIK18051 Δ*araBAD *ϕ(Δ*araEp *P_cp13_-*araE*) Δ*(araFGH)*::*kan903*	P1 from BW27269 to BIK18255
BIK18260	BIK18046 Δ*araBAD *ϕ(Δ*araEp *P_cp13_-*araE*) Δ*(araFGH)*	BIK18256 Km^s ^with pCP20
BIK18261	BIK18051 Δ*araBAD *ϕ(Δ*araEp *P_cp13_-*araE*) Δ*(araFGH)*	BIK18258 Km^s ^with pCP20
BIK18282	BIK18260 Δ*recA*::*kan*	P1 from BIK18120 to BIK18260
BIK18284	BIK18261 Δ*recA*::*kan*	P1 from BIK18120 to BIK18261
BIK18286	BIK18260 Δ(*recB-recC*)::*kan*	P1 from BIK18116 to BIK18260
BIK18288	BIK18261 Δ(*recB-recC*)::*kan*	P1 from BIK18116 to BIK18260
BIK18290	BIK18260 Δ*recA*	BIK18282 Km^s ^with pCP20
BIK18291	BIK18261 Δ*recA*	BIK18284 Km^s ^with pCP20
BIK18292	BIK18260 Δ(*recB-recC*)	BIK18286 Km^s ^with pCP20
BIK18293	BIK18261 Δ(*recB-recC*)	BIK18288 Km^s ^with pCP20
DH5α	F^- ^λ^- ^ϕ 80 *dlacZ *Δ*M*15Δ(*lacZYA-argF*)*U169 deoR*	Laboratory collection [91]
	*recA1 endA1 hsdR17 phoA supE44 thi-1 gyrA96 relA1*	
DH5α MCR	DH5α Δo(*mrr-hsdRMS-mcrBC*)	S Ohta [92]
DH10B	F^- ^*araDJ39 *Δ(*ara*, *leu*)7697 Δ*lacX74 galU galK rpsL*	Laboratory collection [92]
	*deoR *ϕ 80 *dlacZ*Δ*M*15 *endA1 nupG recAl mcrA*	
	Δo(mrr-hsdRMS-mcrBC)	
JWK1944_2	*lacI*^q^*rrnB3 *Δ*lacZ4787 hsdR514 *Δ*(araBAD)567 *Δ*(rhaBAD)568 *Δ*dcm*::*kan*	National Institute of Genetics [116]
BIK18308	DH10B Δ*dcm*::*kan*	P1 from JW1944-2 to DH10B
BMH71-18 *mutS*	Δ(lac-proAB) *supE thi-1 mutS215*::Tn10/F' [*traD36 proAB*^+ ^*lacI*^q ^*lacZΔM15*]	TaKaRa Bio
JC8679	F^- ^λ^- ^*supE44 thr-1 ara-14 leuB6 *Δ(*gpt-proA*)*62 lacY1*	AJ Clark [117]
	*tsx-33 galK2 hisG4 rfbD1 mgl-51 rpsL31 kdgK51 xyl-5*	
	*mtl-1 argE3 thi-1 recB21 recC22 sbcA23*	
BIK1421	JC8679 *mutS215*::Tn10	P1 from BMH71-18 *mutS *to JC8679
GW2730	t*hr-1 leu-6 his-4 argE-3 galK2 strA31 ilvts tif-1 sfiA11*	GC Walker [118]
	Δ*lacU169 lexA71*::Tn5	
BIK1016	MC1060 (pCHR38)	C Sasakawa [119]
BIK1185	GW2730 but *lexA71*::Tn5-Gm	Central part of Tn5 in GW2730 was replaced by Gm
		BIK1016 × GW2730
GC2597	*sfiA*::Tn5 *pyrD thr leu his lac gal malB srl*::Tn10	National Institute of Genetics [120]
	*sfiC str*	
BIK1218	JC8679 *lexA3*(Ind^-^) *malF*::Tn10	N Takahashi [121]
BIK18262	BIK18260 *mutS215*::Tn10	P1 from BIK1421 to BIK18260
BIK18264	BIK18261 *mutS215*::Tn10	P1 from BIK1421 to BIK18261
BIK18270	BIK18260 *malF*::Tn10	P1 fromBIK1218 to BIK18260
BIK18271	BIK18260 *lexA3*(Ind^-^) *malF*::Tn10	P1 fromBIK1218 to BIK18260
BIK18275	BIK18261 *malF*:: Tn10	P1 fromBIK1218 to BIK18261
BIK18276	BIK18261 *lexA3*(Ind^-^) *malF*::Tn10	P1 fromBIK1218 to BIK18261
BIK18266	BIK18260 *sulA*::Tn5	P1 from GC2597 to BIK18260
BIK18268	BIK18261 *sulA*::Tn5	P1 from GC2597 to BIK18261
BIK18278	BIK18260 *sulA*::Tn5 *lexA71*::Tn5-Gm	P1 fromBIK1185 to BIK18266
BIK18280	BIK18261 *sulA*::Tn*5 lexA71*::Tn5-Gm	P1 fromBIK1185 to BIK18268

Gm, gentamycin-resistance gene; *kan*, kanamycin-resistance gene; Km^s^, kanamycin-sensitive; Tc^S^, tetracycline-sensitive.

Progress in genome methylation was measured, in the *mcrBC*-negative strain, by resistance to PvuII cleavage *in vitro *(Figure 4a). The cleaved band pattern shows that the rate of progress of chromosomal DNA methylation after induction correlates with the concentration of arabinose (Figure 4a). The lower (0.0002%) concentration resulted in a delay in methylation of approximately 30 minutes compared to the higher (0.002%) concentration.

We also followed methylation of a single PvuII site on a multi-copy plasmid (pEF60 in Table 1) included in the cell. Plasmids were extracted from cells (BIK18260) harbouring pEF60 and pEF24 (inducible M.PvuII gene) and digested *in vitro *with PvuII and HindIII, which cuts pEF60 at a single site. Quantification of the bands showed that the PvuII site was completely methylated 30 minutes and 60 minutes after induction with 0.002% and 0.0002% arabinose, respectively (data not shown). The time to achieve 50% methylation was about 13 minutes for the higher concentration and about 38 minutes for the lower concentration. They differed by 25 minutes. Thus, the methylation observed with the plasmid agreed well with that observed with the chromosome.

We also observed a low level of PvuII methylation of pEF60 under the repression conditions: 4.1% and 4.3% in one experiment and 5.3% and 6.0% in another; 5% corresponds to 89 sites out of 1,778 PvuII sites in the chromosome of MG1655. This indicates that PvuII methyltransferase is expressed at a low level due to slight leakage from the BAD promoter. This is consistent with earlier reports on this promoter [51,53] and the difficulty in maintaining restriction enzyme genes under this promoter in the repressed state in *E. coli *[54] (M Watanabe, F Khan, Y Furuta and I Kobayashi, unpublished observation).

The induction of PvuII methyltransferase indeed caused immediate chromosome breakage as detected by pulsed-field gel electrophoresis in the *mcrBC*^+ ^strain (Figure 4b) but not in the *mcrBC*^- ^strain (data not shown). With the higher arabinose concentration, huge linear DNA molecules (at the middle point between the well and the 485 kb marker) became prominent by 15 minutes after the induction, and then they appeared to gradually shift into smaller fragments. With the lower arabinose concentration, the huge linear DNA molecules appeared 30 minutes after the induction and decayed in the same way. The chromosome breakage observed thus correlated well with the progress of methylation in the *mcrBC*^- ^strain. Quantification of the DNAs in the well, which likely represent relatively intact chromosomes, revealed that they decreased over time after induction (Figure 4c). These decreases at the different arabinose concentrations correlated well with the progress of methylation in the *mcrBC*^- ^strain.

**Figure 5:**
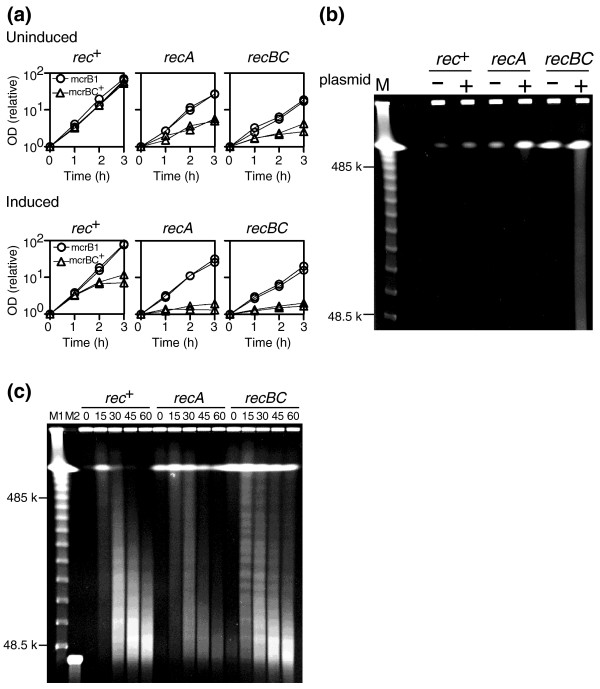
Effect of *recA *and *recBC *mutations on cell growth and chromosome changes. **(a) **Cell growth. BIK18260 (*mcrB1*), BIK18261 (*mcrBC*^+^), BIK18290 (*mcrB1 ΔrecA*), BIK18291 (*mcrBC*^+ ^*ΔrecA*), BIK18292 (*mcrB1 ΔrecBC*) and BIK18293 (*mcrBC*^+^*ΔrecBC*), carrying pEF24 (pSC101::*pvuIIM*, see Table 1), were grown in LB broth with 0.2% glucose and selective antibiotics to exponential phase, diluted to OD600 = 0.1 and further grown with or without 0.0002% arabinose. OD600 was monitored at the indicated time intervals after addition of arabinose. Each value was divided by the value at time zero. **(b) **Chromosomes in uninduced cells. BIK18261 (*mcrBC*^+^), BIK18291 (*mcrBC*^+ ^*ΔrecA*), and BIK18293 (*mcrBC*^+ ^*ΔrecBC*), and their derivatives carrying pEF24 (pSC101::*pvuIIM*) were grown in LB broth with 0.2% glucose and selective antibiotics to exponential phase. Chromosomal DNA was prepared and subjected to pulsed-field agarose gel electrophoresis. M, λ DNA ladder. **(c) **Chromosomes after induction. Chromosome DNA in BIK18261 (*mcrBC*^+^), BIK18291 (*mcrBC*^+ ^*ΔrecA*), and BIK18293 (*mcrBC*^+ ^*ΔrecBC*), carrying pEF24 (pSC101::*pvuIIM*) after induction of PvuII methyltransferase with 0.002% or 0.0002% arabinose. At the indicated time intervals after induction, chromosomal DNA was prepared and subjected to pulsed-field agarose gel electrophoresis. M1, λ DNA ladder; M2, λ DNA cut with HindIII.

The chromosome breakage was accompanied by a decrease in viable cell counts (colony forming units; Figure 4d). The progress of death was again related to the arabinose concentration. The stronger induction led to cell death within 15 minutes, while the weaker induction allowed maintenance of viability for 30 minutes. Many cells appeared as filaments with multiple nuclei or no nucleus (Figure 4e). Inhibition of cell growth as measured in OD was also observed in the *mcrBC*^+ ^cells 1-2 h after induction (Figure 5a, lower left), but not in the repressed state (Figure 5a, upper left).

These results demonstrate a correlation between genome methylation, chromosome breakage, and cell death upon induction of PvuII methyltransferase. They strongly suggest that chromosomal sites methylated by PvuII methyltransferase are cleaved by McrBC and that this cleavage leads to cell death.

### Effect of mutations in DNA-related genes

If the chromosomal sites methylated by PvuII methyltransferase are cleaved by McrBC and this cleavage leads to cell death, mutations in enzymes involved in DNA-related processes might affect these processes. We examined cell growth and chromosome changes in several mutants altered in DNA metabolism in a variety of ways.

RecBCD enzyme is involved in exonucleolytic degradation of DNA from a double-stranded break and generates a recombinogenic single-stranded DNA end [55]. When bound to this single-stranded DNA generated by RecBCD or other enzymes, RecA protein initiates homologous pairing for recombination repair. RecA bound to single-stranded DNA also induces SOS genes through cleavage of their LexA repressor [56]. If RecA and RecBCD are involved in processing and repair of the McrBC-mediated chromosome breakage, their removal might affect cell survival and chromosome processing.

Mutational removal of the host RecBCD/RecA exonuclease/recombinase machinery affected growth not only in the induced state but also in the repressed state (Figure 5a). A likely explanation for the uninduced state is chromosome methylation by slight expression of PvuII methyltansferase (see above). We analyzed chromosomes by pulsed-field gel electrophoresis in strain pairs with and without the P_BAD_-*pvuIIM *plasmid in the *mcrBC*^+ ^background. Our results shown in Figure 5b clearly indicate plasmid-dependent degradation (smear) in the *recBC *mutant and plasmid-dependent increase of huge linear DNAs (the thick band in the midpoint between the well and the 485 kb marker) in the *recA *mutant. These results strongly suggest that partial chromosome methylation led to McrBC-mediated chromosome breakage and that RecBCD/RecA machinery repairs this breakage. The defects in the repair of the McrBC-mediated chromosome breakage are likely the cause of the delayed growth of the *recA *and *recBC *mutants (Figure 5a).

When the methyltransferase is induced, the RecBC/RecA mediated break repair presumably delays growth arrest (Figure 5a). The *recA *or *recBC *mutations slightly affected the loss of cell viability 30 minutes after the induction of methyltransferase (Table 3). However, the final viability level on exposure of the genome to methylation was similar to that in the *rec*^+ ^strain (data not shown).

**Table 3:** Viability loss in various mutants after methyltransferase induction

	Viability (relative)	
	
*E. coli *strain	0% arabinose	0.0002% arabinose
*rec*^+^	2.5, 2.3	1.9, 0.92
Δ*recA*	1.3, 1.7	0.45, 0.31
Δ*recBC*	1.3, 1.2	0.43, 0.59
*lexA*(Ind^-^)*malF*^-^	3.1, 2.5	0.21, 0.15
*malF*^-^	2.1, 2.1	0.85, 0.88
*lexA*(Def)*sulA*^-^	2.1, 2.0	0.96, 0.99
*sulA*^-^	2.1, 2.0	1.4, 1.2
*mutS*^-^	2.0, 1.8	1.4, 1.2

Viability of several mutant *E. coli *strains after induction of PvuII methyltransferase was measured. The number of viable cells was monitored 30 minutes after addition of the lower concentration (0.0002%) of arabinose in two independent experiments. Each value was divided by the value at time zero.

The chromosomes in these mutants showed changes consistent with the above growth patterns and their known properties (Figure 5c). The *recBC *mutant showed a large amount of huge broken chromosomes in the uninduced state; these remained abundant as long as 60 minutes after induction. In the lower area, which corresponds to smaller broken chromosomes, many discrete bands are visible in the *recBC *mutant. This is consistent with the process in which the chromosomes broken by McrBC endonuclease were further degraded by RecBCD exonuclease. The *recA *mutant, unlike the *rec*^+ ^strain, showed more of the huge broken chromosomes even in the uninduced state. In the *rec*^+ ^strain, this species became prominent only 15 minutes after induction and disappeared. In the *recA *mutant, it remained abundant for 30 minutes but started decreasing by 45 minutes after induction. The amount of smaller broken chromosomes in the *recA *strain was less than that in the *rec*^+ ^strain, presumably due to degradation by RecBCD enzyme. No discrete bands are visible in the *recA *mutant, which is consistent with rapid and extensive DNA degradation by RecBCD enzyme. Discrete bands are seen in the *rec*^+ ^strain but they are not so many as in the *recBC *mutant.

These electrophoresis patterns are consistent with the steps of McrBC-mediated chromosomal breakage, RecBCD-mediated exonucleolytic degradation from the break, and RecA-mediated homologous pairing for repair. The RecBCD/RecA-mediated repair was also found for post-segregational killing by a type II RM system [28]. From the results presented in Figure 5 and Table 3, we inferred that the RecBCD/RecA-mediated recombination repair can counteract McrBC's lethal action to some extent at a low methylation level. However, chromosome repair by them appears unable to contribute to cell survival when the genome methylation and the McrBC-mediated cleavage become extensive. This is similar to the chromosome cleavage by a mutant EcoRI enzyme [57,58].

**Figure 6:**
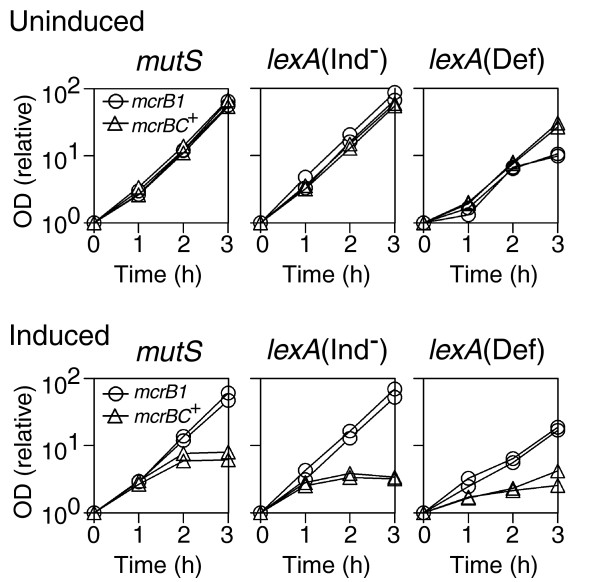
Effect of SOS-related mutations on cell growth. BIK18262 (*mcrB1 mutS*), BIK18264 (*mcrBC*^+^*mutS*), BIK18271 (*mcrB1 lexA*(Ind^-^)), BIK18276 (*mcrBC*^+^*lexA*(Ind^-^)), BIK18278 (*mcrB1 lexA*(Def)), BIK18280 (*mcrBC*^+ ^*lexA*(Def)), carrying pEF24 (pSC101::*pvuIIM*; see Table 1), were grown in LB broth with 0.2% glucose and selective antibiotics to exponential phase, diluted to OD600 = 0.1 and further grown with or without 0.0002% arabinose. OD600 was monitored at the indicated time intervals after addition of arabinose. Each value was divided by the value at time zero.

The RecA/RecBCD function is also involved in the SOS response as mentioned. The cell filamentation was not observed in a *recA *deletion strain (data not shown). This indicates that the cell filamentation we observed represents an SOS response. In order to assess the effects of the SOS response on McrBC-mediated growth inhibition and cell death, we examined SOS-related mutants (Figure 6 and Table 3). Among these, the *lexA*(Ind^-^) mutant is defective in SOS induction, the *lexA*(Def) mutant is constitutive for SOS induction, and the *mutS *mutant shows less background DNA breaks under some genetic backgrounds [59].

These mutants showed McrBC-dependent growth inhibition when M.PvuII was induced, but not in the repressed state (Figure 6). McrBC-mediated inhibition observed in the *lexA*(Ind^-^) mutant was stronger than that in the *rec*^+ ^strain but not so strong as in the *recA *strain (Figure 5a). A simple interpretation of this result is that the defect in repair in the *recA*-negative mutant cannot be entirely attributed to the absence of the SOS response. In other words, RecA is likely to play a direct role, presumably, in recombination repair. The *lexA*(Ind^-^) strain also showed severe loss of cell viability 30 minutes after induction (Table 3). The results with *lexA*(Def) are difficult to interpret because the *lexA*(Def) *mcrB1 *strain showed slow growth. It is known that *lexA*(Def) mutation delays growth even in the *sulA*-negative background [60]. This effect could be exaggerated with McrBC-mediated chromosome breakage upon genome methylation. The *mutS *mutant was indistinguishable from the *rec*^+ ^(*mutS*^+^) strain in these measurements. From these results, we inferred that the SOS response and RecA/RecBCD-mediated DNA recombination/repair both affect cell death/survival upon McrBC action on the methylated genome. The repair systems, however, cannot block cell death upon extensive chromosome methylation and cleavage. These observations are consistent with our hypothesis that chromosome methylation leads to its McrBC-mediated lethal cleavage.

### Generality and specificity of McrBC action against DNA methyltransferases

**Figure 7:**
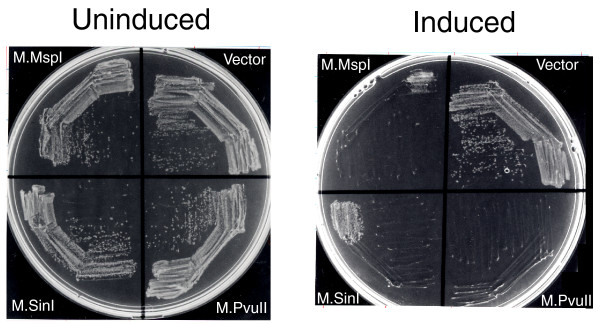
McrBC-mediated cell death with DNA methyltransferases. Cells (BIK18308) harboring pEF46 (P_BAD_-*mcrBC*; see Materials and Methods) and pEF43 (M.PvuII), pSI4 (M.SinI), pNW106RM2-3 (M.MspI), or pBAD30 (vector) were streaked on LB agar plate containing 30 μg/ml chloramphenicol and 25 μg/ml ampicillin, and 0.2% glucose or 0.2% arabinose. Plates were incubated overnight at 37°C.

In order to investigate the generality and specificity of McrBC-mediated cell death with regard to DNA methyltransferase specificity, we expressed McrBC in a cell carrying one of several methyltransferases with different specificities. First, *mcrBC *of *E. coli *was placed under the P_BAD _promoter (pEF46 in Table 1). As expected, McrBC induction in a cell harboring another plasmid encoding M.PvuII (CAG^m4^CTG) led to cell death in the colony formation assay (Figure 7). McrBC induction also led to cell death with M.SinI (GGW^m5^CC) and M.MspI (^m5^CCGG) (Figure 7) but not with M.SsoII (C^m5^CNGG) (data not shown). These results are consistent with the R^m^C sequence specificity of McrBC observed *in vitro *[33]. Our interpretation is that McrBC has the potential to act as a defense system against many DNA methyltransferases of an appropriate specificity.

### Molecular evolutionary analyses of McrB and McrC reveal their frequent loss and horizontal transfer between distantly related genomes

The above experimental results provide an answer to the question we first formulated. It is very likely that McrBC cleaves host chromosomes and causes cell death upon genome methylation and that this cell death inhibits propagation of the methyltransferase gene (Figure 1b). McrBC was also demonstrated to severely restrict bacteriophages carrying hydroxymethylated C in place of C in their genomes [34,35,61,62]. Which of these actions of McrBC has been providing selective advantage for their spread and maintenance during evolution?

In order to address this question, we focused on the similarity of McrBC with type II RM systems in the action of host killing by chromosome cleavage. As illustrated in Figure 1a, when a type II RM gene complex is replaced by a competitor genetic element, its product restriction enzyme will cleave host chromosomes in which methylation decreases and kill the host (Figure 1a) [22]. This leads to survival of cells retaining the RM gene complex but not its competitor. The McrBC system may likewise contribute to exclusion of epigenetic methylation systems (Figure 1b). A contrast between them is that McrBC action follows gain of methylation, as opposed to loss of methylation.

The potential for host killing by type II RM systems indicates their relative independence from the host. They act as a unit of selection and, in this regard, they might be similar to viral genomes, transposons and other selfish mobile elements. Indeed, there are now many lines of evidence for the mobility of type II RM systems [21]. These include molecular evolutionary evidence for their extensive horizontal transfer between distantly related prokaryotes, carriage by mobile elements such as plasmids and linkage with mobility-related genes. Likely due to this mobility, in addition to the ability to cut incoming DNAs and to fight against competing elements by host killing, type II RM systems are widespread throughout Prokaryota. They are often lost from a genome by various mutations [21]. They are quite diversified in sequence recognition because of frequency-dependent selection in defense against incoming DNAs [63] and/or because of mutual competition for recognition sequence in host killing [18]. We asked whether McrBC homologs show similar properties. If they do so, we might take it as evidence supporting the hypothesis that McrBCs have evolved for their ability to kill the host cell in competition with genome methylation systems and behave as selfish mobile elements.

In order to address these points and evaluate the above two hypotheses for McrBC, we examined its evolutionary history. Using the sequence of McrB and McrC from *E. coli *as queries for PSI-BLAST [64] searches, we identified 199 homologous McrBC-like systems, typically comprising operons with an *mcrB*-like gene followed by a *mcrC*-like gene (see also below). These homologs of the McrBC system are widely distributed in Bacteria and Archaea (Table S1 in Additional data file 1), like, for example, type I or type II RM systems [17]. If *mcrBC *homologs show a very narrow distribution and this correlates with distribution of phages with hydroxymethyl C, the phage defense hypothesis might be favored. We address these issues in the Discussion.

Phylogenetic trees calculated from multiple sequence alignments of McrB and McrC sequences (Materials and methods) reveal very similar topologies, suggesting strong co-evolution of these two proteins (Figure S1 in Additional data file 2). Nine bootstrap-supported branches reveal relationships between sequences from different taxons, indicating a very high probability of distant horizontal gene transfer events, which is also a feature of evolution of type II RM systems [15,65]. In the aforementioned cases, McrB and McrC appear to have experienced joint horizontal transfer.

The *mcrBC *gene complex in *E. coli *K12 was suggested to have been acquired recently [61], which is confirmed by our phylogenetic analysis: McrB and McrC from *E. coli *K12 are not found in a branch specific to Proteobacteria (top part of the tree in Figure S1 in Additional data file 2), but in a branch that also includes *Acidobacteria bacterium *Blin 345 (the closest homolog of *E. coli *McrBC), Firmicutes, and Actinobacteria. In general, McrBC subunits from taxons such as Proteobacteria, Actinobacteria, or Firmicutes form numerous intermixed branches in the tree, suggesting multiple horizontal gene transfers followed by vertical dissemination among diverging species and strains. One example of a branch of functionally similar enzymes from completely different taxons is provided by the family of unusual type II RM systems related to McrBC (including LlaI [66], BsuMI [67], LlaJI [68] and their experimentally uncharacterized homologs) that cleave unmethylated DNA and are accompanied by a pair of type IIS DNA methyltransferases to protect against the cleavage of their self-DNA (labelled type II R-like subfamily in Figure S1 in Additional data file 2).

Another feature revealed by the phylogenetic trees is the presence of two strongly diverged subfamilies of McrBC-like systems, one comprising known McrBC (for example, the one from *E. coli *K12) and McrB-like systems (for example, the aforementioned type II enzymes), and the other comprising solely uncharacterized McrBC-like homologs of unknown function, with the McrC-like component defined as uncharacterized protein family DUF524. It is interesting that members of these two subfamilies show nearly perfectly complementary phylogentic distribution, that is, despite their presence in similar taxons, they do not co-occur in one genome (Table S2 in Additional data file 3 and Table S1 in Additional data file 1), which probably reflects some degree of their mutual incompatibility.

The few events of distant horizontal transfer indicated on the phylogenetic trees correspond only to those cases where an McrB (and/or McrC) homolog from one taxon is found to be embedded in a branch comprising a different taxon (for example, *Deinococcus *within Gammaproteobacteria) and where this branch has bootstrap support >50%. This is a very conservative estimation of horizontal gene transfer events. The trees reveal many other cases of branches with mixed taxons, but their bootstrap support is <50%, indicating lack of statistical support for the local tree topology. When we compared the McrB and McrC trees with the 16S rRNA trees calculated for the same set of species (Figure S2 in Additional data file 4), we found numerous disagreements in deep branches, and agreement only in short branches that connect closely related species. This analysis suggests that McrBC systems have been transmitted horizontally numerous times, but of course they have been also inherited vertically by closely related groups of organisms radiating from their common ancestor (for example, by strains of the same species, such as *Streptococcus pneumoniae*, *Campylobacter jejuni*, or *Yersinia pestis*). However, it is very difficult to quantify the rate of distant horizontal transfer by analyzing a tree with a highly variable bootstrap support for different nodes; therefore, we resorted to an independent strategy.

Gojobori and coworkers [69] have published analysis of 116 completely sequenced prokaryotic genomes, in which they calculated an index of potential distant horizontal transfer for all genes, by comparing the frequency of 'words' of pentanucleotide length within each gene with the average word frequency of the entire genome. We have obtained an updated data set for 165 genomes from Dr Nakamura and Dr Gojobori (personal communication). Among these genomes, 29 contain both McrB and McrC homologs (*D. radiodurans *contains one additional McrB homolog). We have analyzed the horizontal transfer index of all genes encoding McrB and McrC homologs and found that 9 McrB-homologous genes (9/30 = 30%) and 10 McrC-homologous genes (10/29 = 35%) exhibit word frequencies that indicate significant likelihood of distant horizontal gene transfer. Thus, in the sample of McrBC systems, for which data are available, approximately one-third appears to have been derived by a recent horizontal gene transfer event from a distantly related group. For the same set of genomes, we also carried out analysis of the horizontal transfer index of genes from two reference 'house-keeping' protein families: RecA and RpoB. We found no members of RecA or RpoB genes in this sample to be predicted as recently transferred.

We found that the McrBC gene complex tends to be lost quite frequently, as no higher-order taxon is found in which all completely sequenced genomes possess this system. Among 567 completely sequenced genomes in which we looked for McrB/C homologs, we found McrB in only 112 cases (19.8%) and McrC in 108 cases (19.0%); McrB and McrC were found together in 107 cases (18.9%). Thus, we conclude that McrBC systems are frequently transmitted by horizontal gene transfer (in addition to regular vertical transfer), but are also very frequently lost. This argues against the hypothesis that they are conserved due only to their utility for defense against phages or other parasites and favors the hypothesis they behave as selfish (host-killing) mobile elements.

### Genomic neighborhood analysis of McrBC systems suggests their mobility and linkage with genome methylation systems

Type II RM gene complexes are often found on mobile elements such as plasmids, phages, integrons and genomic islands [21]. In accord, they are often linked with mobility-related genes such as transposase homologs and integrase homologs. We examined the neighbourhoods of *mcrBC *homologs expecting to find similar genes.

**Figure 8:**
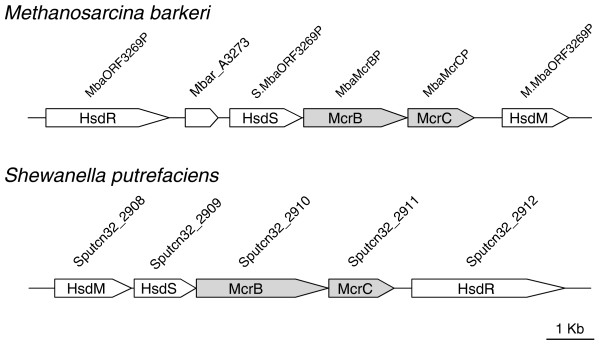
*mcrBC*-like homologs apparently inserted into an RM gene complex. Open reading frame names indicate enzyme names (REBASE) or locus tags (GenBank).

Genomic neighbourhood analysis (Table S2 in Additional data file 3; see Table S1 in Additional data file 1 for the complete data set) revealed that McrB and McrC are tightly linked to each other, suggesting their structure as a single operon. They are frequently associated with homologs of integrases and transposases (Table S2 in Additional data file 3 and Table S1 in Additional data file 1). Several McrBC homologs clearly occur as an insert in an RM gene complex (Figure 8). In addition, eight McrBC-like systems were found on a plasmid (Table S1 in Additional data file 1). These three lines of evidence indicate potential mobility of the *mcrBC *unit. The *mcrBC *homologs were often linked with RM systems or just DNA methyltransferases (Table S2 in Additional data file 3), as first noted for *E. coli *[70]. The implication of this finding is discussed below.

**Figure 9:**
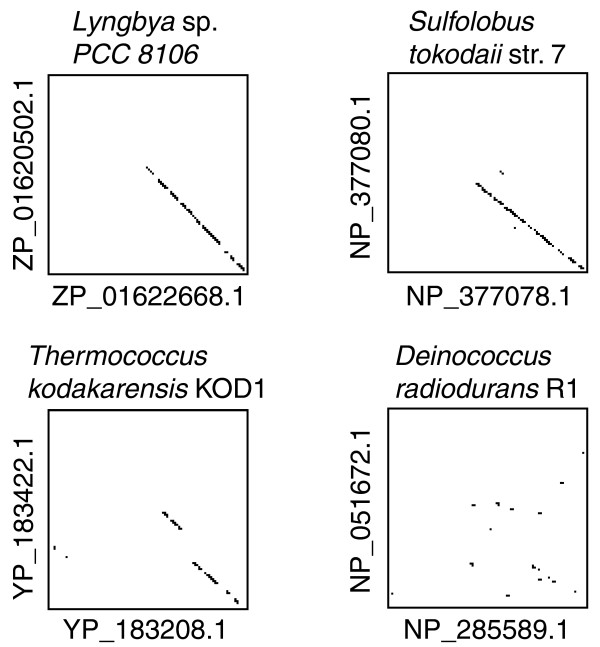
Dot-plot comparison of intragenomic *mcrB *paralogs. Amino acid sequences of a pair of *mcrB *paralogs within one genome were plotted against each other.

Some genomes, such as the *Deinococcus radiodurans *R1 genome, contain two *mcrBC *homologs, sometimes one on a plasmid and the other in the chromosome. Alignment of these pairs of McrB homologs found in the same genome revealed that their amino acid sequences often vary in the amino-terminal region, which is involved in DNA binding [46], suggesting evolutionary shifts in DNA sequence specificity (Figure 9). This parallels the diversity in sequence recognition of type II restriction and modification enzymes.

To investigate the relationship between the diversity of the McrB amino-terminal region and sequence recognition, several McrBC homologs, STOMcrBC (NP_377078.1) and STOMcrBC2 (NP_377080.1) from *Sulforobus tokodaii str. *7, TKOMcrBC (YP_183208.1) and TKOMcrBC2 (YP_183422.1) from *Thermococcus kodakaraensis *KOD1, and DraMcrBC (NP_051672.1) from *D. radiodurans *R1, were amplified from genome DNA and cloned into pBAD30 [51]. These *mcrBC *homologs did not cause cell death in *E. coli *at 37°C in the presence of arabinose in a cell harboring either of the four DNA methyltransferase genes, M.PvuII (CAG^m4^CTG), M.SinI (GGW^m5^CC), M.MspI (^m5^CCGG), or M.SsoII (C^m5^CWGG) (data not shown). EcoKMcrBC from *E. coli *caused cell death sensing genome methylation by M.SinI (GGW^m5^CC) and M.MspI (^m5^CCGG) under the same condition (Figure 7). Therefore, we were unable to link these homologs with the biology of the organisms.

## Discussion

McrBC of *E. coli *can cleave incoming bacteriophage DNAs with methylated bases such as hydroxymethylcytosine [34,35]. This has been thought to be the selective force that allowed their spread and maintenance. In the present work, we propose and examine an alternative (but not necessarily exclusive) hypothesis: when an epigenetic genome methylation system enters a host, McrBC aborts its establishment by cleaving the methylated host genome. We hypothesize that such conflicts with genome methylation systems leading to the sacrificing of a host cell has been the force that allowed their evolution.

In order to examine this hypothesis, we first asked whether such host death through chromosome cleavage in order to exclude DNA methyltransferase genes could take place at all. This is not a trivial question because the genome is protected from cellular DNases by a variety of means (see Background). Our experiments revealed: McrBC-mediated inhibition of establishment of an epigenetic methylation gene (on a plasmid lacking its methylation site; Figure 2); McrBC-mediated chromosome cleavage and degradation following entry of the DNA methyltransferase gene (on a phage genome; Figure 3); a close correlation between genome methylation by the methyltransferase and McrBC-mediated chromosome cleavage, degradation and cell death (Figure 4); and that the effects of mutations in DNA repair-related genes were also consistent with the occurrence of McrBC-mediated lethal chromosome cleavage (Figures 5 and 6). These results strongly argue that the McrBC system can prevent establishment of an epigenetic methylation system by cleaving methylated chromosomes to cause death of the host cell (Figure 1b). The methyltransferase used in our experiments is that of PvuII RM, which was found in a plasmid from a bacterium closely related to *E. coli *and dwelling in the same environment, thus, under conditions that enable horizontal gene transfer. A derivative of this plasmid was demonstrated to be excluded by McrBC (Figure 2b). These results suggest that these experiments are biologically relevant.

Another question is how important has such a capacity of host killing been in evolution. Such a capacity implies that McrBC is in potential conflict with the host genome just as in the case of type II RM systems (Figure 1a). Several type II RM systems kill the host cell when their genes are replaced by a competing element, such as an incompatible plasmid and an allelic gene [20,22,23]. One feature related to such independence from the host genome is the mobility of these RM systems [14]. Just as for type II RM systems, McrBC family members have been shown to be potentially mobile. They have frequently experienced horizontal transfer between distantly related groups, are often linked with mobility-related genes and are widely distributed in Prokaryota. Some of them were found on a plasmid. Their frequent decay is also similar to the decay of type II RM systems [71,72]. These evolutionary and genomic analyses are contrary to the hypothesis that they have been maintained solely as a faithful tool of defense, directly cleaving incoming DNAs, and favor our hypothesis that they have evolved as mobile elements that compete with genome methylation systems through host killing.

How strong is the evidence for the alternative hypothesis of defense against phages with unusual bases, such as T-even phages, by direct cleavage? Phages related to T4 in morphology have been isolated from enterobacterial species closely related to *E. coli *(*Klebsiella*, *Shigella*, and *Yersinia) *and, less frequently, from *Citrobacter*, *Proteus*, *Salmonella*, and *Serratia*. Others propagate on more distantly related bacteria (*Acinetobacter*, *Aeromonas*, *Burkholderia*, and *Vibrios*) [73]. The genomes of the pseudo T-even phage, a subgroup of T4-like phages only distantly related to T4 that includes coliphages and *Aeromonas *phage, can be digested by restriction enzymes [74]. This suggests that only limited nucleotide modifications must be present in their genomes.

Reports of phage genomes with ^5m^C in place of C are rare: one for *Xanthomonas *[75] and the other for *Halobacterium*, an Archaeon [76]. This distribution is in apparent contrast to the wide distribution of McrBC homologs in Prokaryota and could be taken as evidence against the phage defense hypothesis. This evidence is, however, not very strong because we do not know whether there has been an extensive search for phages with unusual bases, ^m^C and others [77], and because we do not know the specificity of most of the McrBC homologs (see below).

Another type IV nuclease, GmrSD, found in an *E. coli *strain targets glucosylated hydroxymethyl-C and may have evolved to cut T4 genome [78]. The resistance of hydroxylmethy-C-containing phage to restriction enzymes but its sensitivity to McrBC [79] and the resistance of glucosylated hydroxymethyl-C to GmrSD but its inhibition by T4-coded internal protein [78] suggest an evolutionary arms race (evolutionary struggle between competing sets of co-evolving genes that develop adaptations and counter-adaptations against each other) between the bacteria and the phage.

The present lines of analyses, combined with examination of the relationships of McrBC homologs with bacteriophages with modified bases in ecological and evolutionary contexts, will help in evaluating these two hypotheses. These two roles may not be necessarily mutually exclusive.

McrBC family members appear to be quite divergent in sequence (Figure S1 in Additional data file 2). Such diversity might be accompanied by diversity in their target recognition. Indeed, members of one McrBC subfamily have been shown to be type II like in that they cleave a specific sequence when unmethylated [66] (Figure S1 in Additional data file 2). The presence of two *mcrB *paralogs diverged in the amino terminus in one genome (Figure 9) is consistent with their divergence in sequence recognition, although our experiments could not demonstrate this. Such divergence in target recognition could also be a basis for the apparent incompatibility of the two subgroups, McrBC-like and the DUF524 subfamily. We imagine that the family of McrBC-like systems may have evolved a variety of substrate specificities to respond to a variety of DNA methylation systems.

Unexpectedly, we found that *mcrBC *homologs are frequently linked with DNA methyltransferase homologs. Many of them are from a type I RM system, while some of them are from a type IIG system (Table 3; Figure 8; Table S1 in Additional data file 1). The linked methyltransferases are expected to have a specificity that does not create a target of the McrBC nuclease. This implies that the McrBC will compete with other methyltransferases of a specificity different from its neighbor (linked) methyltransferase. The base specificity of type I modification enzymes, that is, ^m6^A methylation [80], as opposed to ^m4^C and ^m5^C of McrBC, is consistent with this idea.

Thus, McrBC may be regarded to serve as a player in the competition between different epigenetic genome methylation systems. The insertion of *mcrBC *into a preexisting type I gene complex, as inferred from Figure 8, is explained as acquisition of a helper by the type I system. Such competition processes may have driven diversification of methyltransferases' sequence recognition just as competition between type II RM systems have likely driven diversification of their sequence recognition [18]. Their linkage may also have led to evolution of McrBC-like type II RM systems.

Epigenetic methylation often plays a role in intragenomic conflicts of genetic elements, such as silencing of selfish elements [1]. The present results and the above argument suggest the possibility that epigenetic systems themselves are potentially in a mutual conflict.

A gene programming death of its host has advantages under several conditions. Defense against microbial infection through cell death has been known for animals, plants and prokaryotes [38]. A prokaryotic example of phage exclusion or phage abortion has been known for half a century [39]. Successful infection of a bacterial cell by a phage will lead to production of progeny virus particles, which would then infect the neighboring, likely clonal cells. Then, all the clonal cells and the genes within them might disappear through secondary infection. However, when the first infected cell carries a gene that programs death of the infected cell together with the viral genome, there is no progeny virus production for the secondary infection. The neighboring sibling cells and their genomes would survive. Among these genomes is the gene that programmed the death.

Several type II RM systems trigger cell death when their genes are eliminated by a competitor genetic element [20,22,23] (Figure 1a). There is experimental evidence that one resident type II RM system aborts establishment of another, incoming type II RM system by forcing it to cleave the host genome [19].

Epigenetic genome methylation is involved in transposon silencing [6,81]. There are examples of involvement of other types of epigenetic systems in intragenomic conflicts [1]. The McrBC case is unique in that it directly relates an epigenetic modification to cell death through genome cleavage. To our knowledge, this represents the first report of a defense system against epigenetic systems through cell death.

Mrr, another methyl-specific deoxyribonuclease, induces cell death under high-pressure stress, likely through chromosome breakage [82]. The Mrr gene forms a cassette together with *mcrBC *and the EcoKI type I RM gene complex.

In this article, we treated genes (rather than cells, individual organisms or genomes) as the unit of selection, adopting various strategies to increase their frequency [83]. A gene would increase its frequency if its effects help to do so. This is the basic view in genetics and evolutionary studies, although it might sound anthropomorphic. We use the term 'selfish' as (and only as) 'being a unit of evolutionary selection'. For the situations shown in Figure 1a,b and in programmed death upon infection (see above), expressions such as 'the altruistic cell death is indeed programmed by a selfish gene' are concise and to the point.

The above genes programming death of their host bacterial cell are expected to increase in frequency because of the advantage. However, this argument needs mathematical justification in the domain of evolutionary game theory. The ultimate players of these games must be the genes. For the type of host killing genes illustrated in Figure 1a (addiction or post-segregational killing genes, including type II RM systems), an earlier attempt was unable to demonstrate their spread [84]. This analysis used a model lacking spacial structure, such as a well-mixed liquid culture, where every cell can potentially interact with every other cell. We demonstrated that these genes can increase in frequency if spacial structure is present (that is, if the habitat is structured) so that a cell preferentially interacts with its neighbors [85]. Their increase also depended on the relative cost of the host-killing gene (and its competitor) on the host and on their rate of horizontal transfer.

The *mcrBC *action (Figure 1b) of host killing in competition with the incoming methylation system is formally very similar to this genetic addiction (Figure 1a). We expect that *mcrBC *genes would increase: in the presence of spacial structure (in a structured habitat); if the methylation is costly relative to *mcrBC *genes; and if *mcrBC *genes transfer at a high rate. The second point implies that a methylation system beneficial to the host because of its function (see Background) would not be eliminated. The third point is related to the frequent horizontal transfer of *mcrBC *genes. Mathematical treatment and simulation more specialized to McrBC would help to identify conditions for evolution of this form of programmed cell death and to allow broader interpretations of the role of these genes.

In this work, the term epigenetic indicates 'not genetic but heritable through DNA replication' and is used to distinguish among three modes of DNA methylation: genetic methylation, for example, in the biosynthesis of dTMP from dUMP, then incorporation into DNA by the replication machinery; epigenetic methylation, such as in 5-methylcytosine (^m5^C), N4-methylcytosine (^m4^C) and N6-methyladenine (^m6^A), which is inherited by maintenance methylation after DNA replication; and non-genetic and non-epigenetic methylation as, for example, in O6-methylguanine. It is known that the non-epigenetic and non-genetic DNA methylation in O6-methylguanine triggers cell death [86].

Exogenous expression of mouse DNA methyltransferases induces lethality in *Drosophila *and *Xenopus *[87,88]. The underlying mechanisms and biological significance of such deaths in these heterologous systems remain unclear.

## Conclusion

The observations and considerations presented in this study are consistent with our hypothesis that McrBC-like systems have evolved and are maintained because they would compete with particular epigenetic genome methylation systems by sacrificing their host cell through chromosome cleavage. They can be regarded as selfish mobile elements. This represents, to our knowledge, the first analysis of programmed death machinery protecting the genome from epigenetic systems.

## Materials and methods

### Bacteria and plasmids

All the bacterial strains used were derivatives of *E. coli *K-12 and are listed in Table 2. The Δ*araBAD*, Δ*recA *and Δ*recBC *mutations were introduced into ER1563 [89] using a published procedure [90]. The Δ*araBAD *mutation is a deletion of the *ΔaraBAD *operon and was generated using the H1-ara (GGTTTCGTTTGATTGGCTGTGGTTTTATACAGTCATTACTGCCCGTAATAGTGTAGGCTGGAGCTGCTTC) and H2-araBAD (GGCGTCACACTTTGCTATGCCATAGCATTTTTATCCATAAGATTAGCGGAATTCCGGGGATCCGTCGACC) primers. The Δ*recA *mutation is a deletion of the *recA *gene and was created using the previously described primers [90]. The Δ*recBC *mutation is a deletion from *recB *through *recC *and was generated using the H1-*recBC *(TTCATTACGCCTCCTCCAGGGTCATACCGGCAAACATCTCATCCATCAGGGTGTAGGCTGGAGCTGCTTC) and H2-*recBC *(TCAGGAGCCGCTATGTTAAGGGTCTACCATTCCAATCGTCTGGACGTGCTATTCCGGGGATCCGTCGACC) primers. *E. coli *DH5α [91] and DH5α MCR [92] were used for plasmid construction. Other mutations were introduced by P1 transduction [93].

All the plasmids used are listed in Table 1. A 1,200 bp fragment including the *pvuIIM *gene without the SD sequence was amplified from pYNEC302 [19] using the M.PvuII-1 (5'-GgaattcGAATTCGGGCTGATAAAGGATTT-3') and M.PvuII-2 (5'-GGggtaccGGTACCTTTGCTGAGGCGGTTTT-3') primers. Each PCR primer has an introduced restriction site, for KpnI and EcoRI, respectively, at the 5' end (small letters). The fragment was digested with KpnI and EcoRI and then inserted into pBAD18 [51] to generate pEF1 (P_BAD_-*pvuIIM*; ColE1; Ap). pIK8004 was constructed by Mikihiko Kawai by inserting a NotI linker (GCGGCCGC, TaKaRa Bio, Otsu, Shiga, Japan) into the DraI site of pBR322 (Mikihiko Kawai, personal communication). pEF23 (PBAD-*pvuIIM*; ColE1; Ap) was constructed by ligating a ClaI-SalI fragment of pIK8004 and a ClaI-SalI fragment of pEF1. The pEF24 plasmid (P_BAD_-*pvuIIM*; pSC101; Ap) was constructed by ligating the smaller SmaI-EcoRV fragment of pSC101 and a NotI-SalI fragment of pEF23. pEF30 was constructed by joining the EcoRI-HindIII fragment that contained the BamHI RM gene complex of pYNEC404 to the larger EcoRI-HindIII fragment of pBR322. pEF33 was constructed by eliminating a PvuII site in the *rop *gene of pBR322 by mutation of Ser51 (AGC to AGT). pEF43 was constructed by ligating a KpnI-EcoRI fragment of pEF1 with the larger KpnI-EcoRI fragment in pEF33.

A 2.4 kb fragment including the *mcrB *and *mcrC *gene was amplified from *E. coli *ER1563 using the EcoKMcrBC-for (5'-GGGggtaccATGGAATCTATTCAACCCTGGATTG-3') and EcoKMcrBC-rev (5'-GGGgtcgacTTATTTGAGATATTCATCGAAAATG-3') primers. Each PCR primer has an introduced restriction site for KpnI or SalI at the 5' end (small letters). The fragment was digested with KpnI and SalI and then inserted into pBAD30 [51] to generate pEF46. pEF60 was constructed by deletion of the DraI-StuI fragment, including the ampicillin-resistance gene, through DraI and StuI cleavage followed by self-ligation.

Genomic DNA was obtained from Issei Narumi for *D. radiodurans *R1, Toshiaki Fukui for *T. kodakaraensis*, and Yutaka Kawarabayashi for *S. Tokodaii *str. 7. Other *mcrBC *homologs were similarly amplified from the genomic DNAs using DraMcrBC-for (5'-GGGggtaccATGAGCGACGCTGCCATTTCGTGTT-3') and DraMcrBC-rev (5'-GGGgtcgacTCAGGTCAAGACCGAAGCTGGCCAT-3'), TkoMcrBC-for (5'-GGGggtaccGTGGGCAGATTTGAGATTTCCGAAA-3') and TkoMcrBC-rev (5'-GGGgtcgacTTAAACCTCTCCCGAAGAGCAGAGG-3'), TkoMcrBC2-for (5'-GGGggtaccATGAATCAATCAGTTATAATAGATG-3') and TkoMcrBC2-rev (5'-GGGgtcgacCTAGTTTATTAGCGAATTTAGATAA-3'), StoMcrBC-for (5'-GGGggtaccGTGAACAAAAGAGATATACAACTAC-3') and StoMcrBC-rev (5'-GGGgtcgacTTAGATTTTACGATTTTCGCCTTTT-3'), or StoMcrBC2-for (5'-GGGggtaccGTGAGGTTAAGAAAAAGAGATCTAG-3') and StoMcrBC2-rev (5'-GGGgtcgacTTAACTAATAATACCTTTTTTCTT-3') primers.

A SalI-PstI fragment of pPvuCat16 (pPvu1 ori, *pvuIIM*) and pPvuCat17 (pPvu1 ori) [43] carrying the *cat *gene was replaced by a PCR-generated fragment carrying the *kan *gene from pUC4K to generate pEF65 (pPvu1 ori, *pvuIIM*) and pEF67 (pPvu1 ori), respectively. The *kan *fragment was amplified using kan-for (5'-ACGCgtcgacGTTGTGTCTCAAAATCTC-3') and kan-rev (5'-TTctgcagAACCAATTCTGATTAGAAAA-3') primers.

### Phages

λ phage strain LIK891 was as described [94]. This phage possesses a single site for HindIII located near the *int *gene, a deletion between EcoRI sites 1 and 2, immunity substitution from phage 21 (*imm21*), and deletion between SalI sites, which inactivates the *red *and *gam *genes. λ phage strain LIK891 carries 15 PvuII sites. M.PvuII-modified λ LIK891 was prepared on ER1562 (pYNEC313 = pBR322::*pvuIIRMC*) by the plate lysate method [95], while its unmodified version was prepared on ER1562. λ phage strain LEF1 was constructed by inserting a Hind III fragment of pYNEC301 into the HindIII site of LIK891.

The modification status of the phage was confirmed by resistance to PvuII restriction endonuclease both *in vitro *and *in vivo*. λ phage prepared by the plate method (see above) was purified by ultra-centrifugation [96]. The phage genome DNA was purified from the λ preparation using a λ DNA purification kit (TaKaRa Bio), digested with PvuII (TaKaRa Bio), and subjected to pulsed-field agarose gel electrophoresis. PvuII treatment introduced no detectable change in electrophoresis pattern for PvuII-modified λ LIK891 and LEF1 DNAs when it completely cleaved unmodified λ LIK891 DNA (date not shown). PvuII-modified λ LIK891 and LEF1 showed no decrease in plaque formation efficiency in ER1562 (pYNEC313 = pBR322::*pvuIIRMC*) compared to that in ER1562, although the unmodified λ LIK891 was restricted severely to a relative plaque formation efficiency of 4 × 10^-6^.

For the phage plaque assay, an overnight culture of *E. coli *was diluted 100-fold and grown to mid-exponential phase at 37°C with aeration in λ polypepton broth (Nihon Seiyaku, Chiyoda-ku, Tokyo, Japan) with 0.2% maltose and 10 mM MgSO_4_. Phage was appropriately diluted and mixed with 100 μl of the fresh culture. After incubation at 37°C for 30 minutes, the phage-bacteria complex was mixed with 2 ml of λ polypepton top agar and poured on λ polypepton agar plate. After incubation at 37°C for 18 h, plaques were counted.

### Plasmid preparation and quantitative transformation

Plasmid DNA was purified using a QIAGEN kit (Qiagen, Germantown, MD, USA). To confirm the accuracy of transformation, varying amounts of pUC19 plasmid DNA were transformed into *E. coli *DH5α by electroporation with a Gene Pulser (Bio-Rad, Hercules, California, USA), as described [97]. Various amounts of pACYC184 plasmid were added to give a total DNA amount of 200 ng.

For comparison of plasmids, 100 ng of plasmid DNA, purified by cesium chloride-ethidium bromide centrifugation, was used. The number of transformants was determined by spreading an aliquot on agar plates containing ampicillin (50 μg/ml). Relative transformation efficiency to the vector was calculated to normalize the transformation efficiency between strains.

### Induction of PvuII methyltransferase

Overnight cultures carrying pEF24 (PBAD-*pvuIIM*; pSC101; Ap) were diluted 100-fold and grown at 37°C in Luria-Bertani (LB) medium containing 25 μg/ml ampicillin and 0.2% glucose. When the cultures reached the mid-exponential phase, the cultures were adjusted to OD600 = 0.1 in fresh medium containing 25 μg/ml ampicillin and 0.0002% or 0.002% arabinose. The cultures were appropriately diluted to maintain them at the exponential phase. To measure colony-forming units, cells were diluted in LB with 0.2% glucose and spread on LB agar with 0.2% glucose.

### Preparation of chromosomal DNA

Cells were lysed within an agarose gel by modification of a published method [98] as follows. The cells were mixed with 2,4-dinitrophenol to block energy metabolism at the indicated time intervals (in minutes) after the induction of PvuII methyltransferase. After centrifugation, the pellet was washed twice with suspension buffer (10 mM Tris-HCl (pH 8.0), 20 mM NaCl and 50 mM EDTA). The cells were mixed with an equal volume of the same buffer containing 2% low-melting agarose (SeaPlaque GTG agarose, FMC Bioproducts, Rockland, Massachusetts, USA), pipetted into a plug mold (Bio-Rad), and allowed to cool. The resulting plugs were incubated at 37°C for 2 h in lysozyme solution (lysozyme (1 mg/ml), sodium deoxycholate (0.2%), sodium lauryl sarcosinate (0.5%), 10 mM Tris-HCl (pH 8.0), 50 mM NaCl, 10 mM EDTA). The plugs were then washed twice with sterilized water, incubated at 50°C for 15 h in proteinase K solution (100 mM EDTA (pH 8.0), sodium deoxycholate (0.2%), sodium lauryl sarcosinate (1%) and proteinase K (1 mg/ml)), and washed with wash buffer (20 mM Tris-HCl (pH 8.0) and 50 mM EDTA). For PvuII restriction enzyme digestion, the plugs were washed in 2 mM PMSF (Phenylmethylsulfonyl fluoride) in 10 mM Tris-HCl (pH 8.0) and 1 mM EDTA to inactivate residual Proteinase K and incubated in 500 μl of the 1× M buffer (TaKaRa Bio) and 50 units of PvuII (TaKaRa Bio) per plug at 37°C for 15 h. After incubation, the plugs were washed with the wash buffer.

### Pulsed-field gel electrophoresis

Samples were subjected to pulsed-field gel electrophoresis in a CHEF-DR III System (Bio-Rad) under the following conditions: 18 h or 12 h run time, 5- to 40-s of switch time ramp, 120° included angle, 6 V/cm, 0.5× Tris-borate-EDTA buffer (0.045 M Tris-borate, 0.01 M EDTA), 14°C, 1.2% Certified Megabase agarose (Bio-Rad). For size markers, a λ DNA ladder (Bio-Rad) and λ DNA/HindIII markers were used. After the run, the gel was stained with ethidium bromide for 1 h, destained in water, and examined using a FLA-5100 image analyzer (Fujifilm, Minato-ku, Tokyo, Japan). The fluorescence response of each well was determined using the profile analysis feature of the Image Gauge software (Fujifilm). Background was subtracted.

### Microscopic observation

Cells were mixed with an equal volume of methanol-formaldehyde (2:1). After incubation on ice for 10 minutes, the cells were collected by centrifugation, resuspended in 10 mM Tris-HCl (pH 7.5) and 10 mM MgSO_4 _and stained with DAPI (4',6-diamidino-2-phenylindole dihydrochloride). The cells were observed using a fluorescence microscope.

### Phylogenetic analysis

McrB and McrC homologs were identified by PSI-BLAST [64] searches of the GenBank database. Multiple sequence alignments were constructed by iterating automated alignment construction with MUSCLE [99] and manual correction until all conserved regions had been satisfactorily aligned. Incomplete protein sequences that lacked more than 50% conserved regions have been omitted from further analyses. MEGA4 [100] was used to calculate Minimum Evolution phylogenetic trees of McrB and McrC families for conserved regions with <5% gaps, using the following options: JTT matrix, 1,000 bootstrap replicates, Close Neighbor Interchange level = 2, with initial trees calculated by the neighbor-joining method.

The alignment of 481,650 16S rRNA sequences was obtained from the RDP database [101]. Only one representative sequence per genome (113 sequences total) was retained for further analysis. Missing sequences were retrieved manually from the GenBank database, and subsequently aligned to the partial 16S rRNA alignment from the RDP. The multiple sequence alignment was refined by hand to remove truncated variants. The final alignment comprising 154 16S rRNA sequences was used to calculate the Minimum Evolution tree with MEGA 4.0 (Maximum Composite Likelihood, 1,000 bootstrap replicates). The dot-plot analysis of amino acid sequences was performed by DNASIS (Hitachi Software Engineering, Shinagawa-ku, Tokyo, Japan) with the following parameters: check size = 10, matching size = 6.

### Neighbourhood analysis

The *mcrB *neighborhood has been defined as 10,000 base pairs upstream and 10,000 base pairs downstream of the translation start and stop codons of the *mcrB*-like gene. The corresponding DNA sequences together with the protein sequences encoded within their boundaries were retrieved from GenBank [102]. For all proteins encoded in the McrB neighborhood, the ultra-sensitive HHSEARCH program for detection of homology [103] was used to search for amino acid sequence similarity against the PFAM database of protein families and domains. A membership in a top-scoring protein family was assigned to a given McrB neighbor only for matches with an e-value = 0.001; in all the remaining cases, the sequences have been considered unassigned. Analogous homology assignments have been made for all protein sequences in three representative genomes: *E. coli *K12 [104], *Bacillus subtilis *[105], and *Pyrococcus abyssi *[106].

## Abbreviations

DAPI: 4',6-diamidino-2-phenylindole dihydrochloride; LB: Luria-Bertani; RM: restriction-modification.

## Authors' contributions

EF carried out experiments and initiated neighborhood analysis. KHK carried out evolutionary analyses and expanded neighborhood analysis. JMB guided KHK in these. IK conceived of the study, and participated in its design and coordination. All authors participated in writing and approved the final manuscript.

## Additional data files

The following additional data are available with the online version of this paper. Additional data file 1 is a table (Table S1) listing the genomic contexts of *mcrB *homologs. Additional data file 2 is a figure (Figure S1) of phylogenetic trees of McrB and McrC. Additional data file 3 is a table (Table S2) listing neighbors of *mcrB *homologs. Additional data file 4 is a figure (Figure S2) of a phylogenetic tree of the 16S rRNA gene.

## Supplementary Material

Additional data file 1 Genomic contexts of *mcrB *homologs. Click here for file

Additional data file 2 Phylogenetic trees of McrB and McrC. Click here for file

Additional data file 3 Neighbors of *mcrB *homologs. Click here for file

Additional data file 4 Phylogenetic tree of the 16S rRNA gene. Click here for file
