# Supplementary material for: Cell death upon epigenetic genome methylation: a novel function of methyl-specific deoxyribonucleases
Source: Genome Biol. 2008 Nov 21;9(11):R163. doi: 10.1186/gb-2008-9-11-r163 (PMC2614495; doi:10.1186/gb-2008-9-11-r163)
Supplement: Additional data file 1 — Genomic contexts of mcrB homologs. [file gb-2008-9-11-r163-S1.doc]

## Table S1. Genomic context of *mcrB* homologs.

| Organism | **Length** | McrB | **Accession number** | McrC | Other neighbors | |
| --- | --- | --- | --- | --- | --- | --- |
|  | **(aa)** | GI number | **(NCBI)** | GI number | **Restriction/modification enzyme homologs** | **Transposase/Integrase homologs (type)** |
| **Bacteria** |  |  |  |  |  |  |
| **Proteobacteria** |  |  |  |  |  |  |
| *Azoarcus sp. EbN1* | 353 | 56479205 | YP_160794.1 | 56479206 | ND^*^ | Transposase x 2 |
| *Azoarcus sp. EbN1* | 820 | 56478043 | YP_159632.1 | 56478044 | ND | ND |
| *Burkholderia phymatum STM815* | 798 | 118031290 | ZP_01502745.1 | 118031338 | ND | ND |
| *Burkholderia pseudomallei 668* | 666 | 126438633 | YP_001060513.1 | ND | M.Bps668ORF3504P, V.Bps668ORF3504P | Integrase |
| *Burkholderia sp. 383* | 696 | 78060551 | YP_367126.1 | 78060552 | ND | ND |
| *Campylobacter concisus 13826* | 412 | 157165780 | YP_001466263 | 157164216 | M.HaeIII | ND |
| *Campylobacter concisus 13826* | 590 | 157164852 | YP_001466276 | 157164216 | ND | ND |
| *Campylobacter jejuni RM1221* | 662 | 57237146 | YP_178158.1 | 57237147 | ND | ND |
| *Campylobacter jejuni subsp. doylei 269.97* | 598 | 145958530 | ZP_01807491.1 | 145958531 | ND | ND |
| *Campylobacter jejuni subsp. jejuni 81-176* | 603 | 121612502 | YP_999863.1 | 121613370 remote^¶^ | ND | ND |
| *Campylobacter jejuni subsp. jejuni 84-25* | 591 | 88597447 | ZP_01100682.1 | 88597234 remote | ND | ND |
| *Campylobacter jejuni subsp. jejuni CF93-6* | 452 | 86149519 | ZP_01067749.1 | 86149657 remote | ND | ND |
| *Campylobacter jejuni subsp. jejuni CG8486* | 531 | 145844702 | EDK21807.1 | 145844703 | ND | ND |
| *Campylobacter jejuni subsp. jejuni HB93-13* | 773 | 86153849 | ZP_01072052.1 | 86153658 remote | ND | ND |
| *Campylobacter jejuni subsp. jejuni NCTC 11168* | 783 | 15791527 | NP_281350.1 | 15791528 | ND | ND |
| *Campylobacter lari RM2100* | 638 | 57240286 | ZP_00368235.1 | 57240285, 57240758 remote | ND | ND |
| *Chromobacterium violaceum ATCC 12472* | 670 | 34495461 | NP_899676.1 | 34495462 | ND | Integrase |
| *Congregibacter litoralis KT71* | 507 | 88698968 | EAQ96086.1 | 88706786 | ND | Transposase |
| *Escherichia coli B171* | 549 | 75212130 | ZP_00712170.1 | ND | REase^‡^, HsdR, HsdS, HsdM | Integrase, Transposase (inactive) x 3 |
| *Escherichia coli E24377A* | 685 | 157155277 | YP_001464336.1 | 157155806 | MTase^†^ | Integrase, Transposase (inactive) x 9 |
| *Escherichia coli HS* | 691 | 157163750 | YP_001461068.1 | 157163749 | ND | Integrase x 2, Transposase (inactive) x 2 |
| *Escherichia coli K12* | 459 | 90111737 | NP_418766.4 | 16132166 | S.EcoKI,M.EcoKI,R.EcoKI | ND |
| *Escherichia coli W3110* | 459 | 89111055 | AP_004835.1 | 89111054 | S.EcoW3110ORF4339P, M.EcoW3110ORF4339P, EcoW3110ORF4339P, EcoW3110MrrP | Transposase |
| *Geobacter uraniumreducens Rf4* | 454 | 148266126 | YP_001232832.1 | 148266127 | ND | ND |
| *Hahella chejuensis KCTC 2396* | 777 | 83645100 | YP_433535.1 | 83645099 | ND | ND |
| *Helicobacter acinonychis str. Sheeba* | 447 | 109946970 | YP_664198.1 | 109946971 | M.HacSORF360P | ND |
| *Helicobacter hepaticus ATCC 51449* | 670 | 32266942 | NP_860974.1 | 32266941 | ND | ND |
| *Helicobacter pylori 26695* | 518 | 15645080 | NP_207250.1 | ND | ND | IS*1* Transposase |
| *Helicobacter pylori J99* | 448 | 15611234 | NP_222885.1 | 15611235 | ND | ND |
| *Idiomarina baltica OS145* | 678 | 85711475 | ZP_01042533.1 | 85711476 | HsdM, HsdS, HsdR | Phage Integrase x 2 |
| *Janthinobacterium sp. Marseille* | 541 | 151283216 | ABR91626.1 | 151283215 | MTase | ND |
| *Marinobacter aquaeolei VT8* | 751 | 120552972 | YP_957323.1 | 120552971 | ND | Integrase |
| *Marinomonas sp. MWYL1* | 636 | 152998368 | YP_001343203.1 | 152998367 | MTase | ND |
| *Nitrobacter hamburgensis X14* | 734 | 92109628 | YP_571915.1 | 92109629 | NhaXORF4499P | Integrase, phage Integrase x 2 |
| *Nitrosomonas europaea ATCC 19718* | 740 | 30250447 | NP_842517.1 | 30250448 | M.NeuORF2522AP, M.NeuORF2522PB, S.NeuORF2522P, NeuORF2522P | Integrase x 3, Transposase (IS*911*) x 2 |
| *Photobacterium profundum SS9* | 599 | 54307416 | YP_128436.1 | 54307417 | ND | ND |
| *Plesiocystis pacifica SIR-1* | 750 | 149920792 | ZP_01909255.1 | ND | MTase, HsdM, HdsS, HsdR | ND |
| *Polaromonas naphthalenivorans CJ2* | 778 | 121582843 | YP_973285.1 | ND | ND | ND |
| *Pseudomonas entomophila L48* | 675 | 104782352 | YP_608850.1 | 104782351 | ND | ND |
| *Pseudomonas fluorescens PfO-1* | 678 | 77458885 | YP_348391.1 | 77458886 | ND | ND |
| *Pseudomonas putida F1* | 862 | 148549822 | YP_001269924.1 | 148549821 | ND | ND |
| *Pseudomonas syringae pv. syringae B728a* | 517 | 66046953 | YP_236794.1 | 66046952 | ND | ND |
| *Pseudomonas syringae pv. tomato str. DC3000* | 517 | 28868859 | NP_791478.1 | 28868860 | ND | Transposase, Transposase (IS*Psy4*) |
| *Psychrobacter arcticus 273-4* | 498 | 71064806 | YP_263533.1 | 71064807 | ND | ND |
| *Psychrobacter cryohalolentis K5* | 810 | 93005767 | YP_580204.1 | 93005768 | PcrKORF952P, M.PcrKORF952P, S.PcrKORF952P | Transposase (IS*3*/IS*911*), Transposase (IS*30*) x 2, Transposase (mutator) x 2, Transposase |
| *Psychrobacter cryohalolentis K5* | 498 | 93005083 | YP_579520.1 | 93005084 | ND | ND |
| *Psychrobacter sp. PRwf-1* | 655 | 148653161 | YP_001280254.1 | 148653155 separated from McrB by transposases and integrases (8 total in the region) | ND | Transposase x4,  Integrase x4 |
| *Psychrobacter sp. PRwf-1* | 481 | 148653828 | YP_001280921.1 | 148653827 | ND | ND |
| *Ralstonia eutropha H16* | 792 | 113866039 | YP_724528.1 | 113866040 | M.ReuHORF4P, S.ReuHORF4P, ReuHORF4P | ND |
| *Ralstonia metallidurans CH34* | 303 | 94311600 | YP_584810.1 | 94311599 | M.RmeAORF2670P | ND |
| *Rhodopseudomonas palustris BisB5* | 450 | 91976450 | YP_569109.1 | 91976451 | M.RpaB5ORF1972P | ND |
| *Rhodopseudomonas palustris HaA2* | 834 | 86748715 | YP_485211.1 | 86748716 | ND | ND |
| *Roseovarius sp. TM1035* | 672 | 149204092 | ZP_01881060.1 | ND | ND | Integrase,Transposase |
| *Salmonella enterica subsp. enterica serovar Choleraesuis str. SC-B67* | 735 | 60115676 | YP_209467.1 | 60115677 | S.MaqORF31P, M.MaqORF31P, | Integrase,Transposase, IS*1* Integrase |
| *Shewanella baltica OS185* | 605 | 153000505 | YP_001366186.1 | 153000506 | HsdM, HsdS, HsdR | ND |
| *Shewanella putrefaciens CN-32* | 900 | 146294002 | YP_001184426.1 | 146294003 | HsdM, HsdS, HsdR | ND |
| *Shewanella woodyi ATCC 51908* | 755 | 118073093 | ZP_01541277.1 | 118073094 | MTase | ND |
| *Shewanella woodyi ATCC 51908* | 712 | 118071309 | ZP_01539505.1 | 118071310 | R.SdeODORF1235P, M.SdeODORF1235P, S.SdeODORF1235P | ND |
| *Stenotrophomonas maltophilia R551-3* | 449 | 119878261 | ZP_01645211.1 | 119878262 | ND | ND |
| *Vibrio cholerae RC385* | 379 | 116217130 | ZP_01482944.1 | 116217131 | ND | ND |
| *Vibrio cholerae V51* | 688 | 125621518 | EAZ49851.1 | 125621517 | HsdS, HsdM, HsdR | ND |
| *Vibrio parahaemolyticus RIMD 2210633* | 835 | 28898916 | NP_798521.1 | 28898917 | ND | Integrase |
| *Vibrio splendidus 12B01* | 829 | 84390144 | ZP_00991406.1 | 84390145 | HsdR, HsdM, HsdS | Transposase |
| *Xanthomonas campestris pv. campestris str. 8004* | 842 | 66767707 | YP_242469.1 | 66767708 | ND | ND |
| *Xanthomonas campestris pv. campestris str. ATCC 33913* | 842 | 21232164 | NP_638081.1 | 21232163 | ND | ND |
| *Yersinia frederiksenii ATCC 33641* | 687 | 77974092 | ZP_00829635.1 | 77974093 | HsdM, HsdS, HsdR | Integrase |
| *Yersinia intermedia ATCC 29909* | 651 | 77977894 | ZP_00833332.1 | 77977893 | ND | Transposase (inactive) |
| *Yersinia pestis Angola* | 687 | 77632991 | ZP_00795151.1 | 77632990 | ND | Transposase |
| *Yersinia pestis Antiqua* | 687 | 108809887 | YP_653803.1 | 108809886 | ND | Integrase |
| *Yersinia pestis biovar Microtus str. 91001* | 687 | 45443526 | NP_995065.1 | 45443525 | ND | Integrase |
| *Yersinia pestis biovar Microtus str. 91001* | 687 | 45438395 | AAS63942.1 | 45438394 | ND | Integrase |
| *Yersinia pestis biovar Orientalis str. IP275* | 687 | 89103250 | ZP_01175836.1 | 89103249 | ND | Transposase, Integrase |
| *Yersinia pestis CO92* | 687 | 16120721 | NP_404034.1 | 16120722 | M.YpeORF391P | Transposase, Transposase (IS*100*), Transposase (partial) |
| *Yersinia pestis KIM* | 688 | 22127668 | NP_671091.1 | 22127667 | M.YpeKORF3792P | Transposase x 5 |
| *Yersinia pestis Nepal516* | 687 | 108810424 | YP_646191.1 | 108810425 | ND | Integrase |
| *Yersinia pestis Pestoides F* | 687 | 145600497 | YP_001164573.1 | 145600496 | ND | Integrase, Phage Integrase |
|  |  |  |  |  |  |  |
| **Firmicutes** |  |  |  |  |  |  |
| *Bacillus anthracis str. Ames* | 843 | 30261056 | NP_843433.1 | 30261057 | ND | ND |
| *Bacillus anthracis str. Ames* | 606 | 30262292 | NP_844669.1 | 30262293 | ND | ND |
| *Bacillus anthracis str. Sterne* | 609 | 49185139 | YP_028391.1 | 49185140 | ND | ND |
| *Bacillus anthracis str. Sterne* | 847 | 49183897 | YP_027149.1 | 49183898 | ND | ND |
| *Bacillus cereus ATCC 10987* | 606 | 42781381 | NP_978628.1 | 42781382 | ND | ND |
| *Bacillus cereus ATCC 10987* | 792 | 42780092 | NP_977339.1 | 42780092 (McrB-McrC fusion protein) | M.BceSI, R.BceSI | ND |
| *Bacillus cereus ATCC 10987* | 343 | 4584112 | CAB40609.1 | 4584112 (McrB-McrC fusion protein) | M.BseCI, BseCI | ND |
| *Bacillus cereus ATCC 14579* | 578 | 30019094 | NP_830725.1 | 30019095 | R2.Bce14579ORF939P, M.Bce14579ORF939P | ND |
| *Bacillus cereus E33L* | 609 | 52143175 | YP_083655.1 | 52143173 | ND | ND |
| *Bacillus cereus G9241* | 844 | 47564886 | ZP_00235930.1 | 47564887 | ND | ND |
| *Bacillus cereus G9241* | 592 | 47565642 | ZP_00236682.1 | 47565643 | ND | ND |
| *Bacillus cereus subsp. cytotoxis NVH 391-98* | 605 | 152975458 | YP_001374975.1 | 152975459 | ND | ND |
| *Bacillus coagulans 36D1* | 804 | 124519812 | ZP_01695282.1 | 124519811 | HsdM, HsdS, HsdR | Transposase (IS*4*) |
| *Bacillus coagulans 36D1* | 675 | 124520429 | ZP_01695585.1 | 124520428 | ND | ND |
| *Bacillus sp. NRRL B-14911* | 468 | 89097273 | ZP_01170163.1 | 89097272 | Vsr^§^ | Transposase |
| *Bacillus subtilis subsp. subtilis str. 168* | 343 | 16077677 | NP_388491.1 | 16077678 | ND | ND |
| *Bacillus thuringiensis serovar israelensis ATCC 35646* | 608 | 75762961 | ZP_00742761.1 | 75762960 | ND | ND |
| *Bacillus thuringiensis serovar konkukian str. 97-27* | 609 | 49477591 | YP_036395.1 | 49477593 | ND | ND |
| *Bacillus thuringiensis str. Al Hakam* | 609 | 118477710 | YP_894861.1 | 118477711 | ND | ND |
| *Bacillus thuringiensis str. Al Hakam* | 844 | 118476566 | YP_893717.1 | 118476567 | M.BthAHORF843P, RM.BthAHORF843P | ND |
| *Bacillus weihenstephanensis KBAB4* | 513 | 89205405 | ZP_01183975.1 | 89205404 | MTase | Phage Integrase |
| *Bacillus weihenstephanensis KBAB4* | 605 | 89205563 | ZP_01184132.1 | 89205562 | ND | ND |
| *Caldicellulosiruptor saccharolyticus DSM 8903* | 783 | 146295648 | YP_001179419.1 | 146295649 | ND | Integrase |
| *Carboxydothermus hydrogenoformans Z-2901* | 569 | 78043526 | YP_360547.1 | 78045215 | ND | ND |
| *Clostridium difficile 630* | 646 | 126699711 | YP_001088608.1 | 126699710 | ND | ND |
| *Clostridium difficile QCD-32g58* | 550 | 145954110 | ZP_01803117.1 | 145954108 | ND | ND |
| *Clostridium novyi NT* | 301 | 118444185 | YP_877506.1 | 118443173 | ND | ND |
| *Clostridium perfringens ATCC 13124* | 587 | 110799098 | YP_695439.1 | 110801259 | ND | ND |
| *Desulfotomaculum reducens MI-1* | 629 | 134297904 | YP_001111400.1 | 134297905 | ND | Phage Integrase, Transposase (IS*Chy6*), Transposase (IS*118*/IS*110*/IS*902*), Transposase (IS*111A*, IS*1328*/IS*1533*) |
| *Geobacillus kaustophilus HTA426* | 559 | 56419913 | YP_147231.1 | 56419914 | M.GkaORF1380P, S.GkaORF1380P, GkaORF1380P | ND |
| *Geobacillus thermodenitrificans NG80-2* | 350 | 138894901 | YP_001125353.1 | 138894901 (truncated) | ND | Transposase |
| *Lactococcus lactis ME2* | 337 | 639888 | AAA65069.1 | 639889 | M.LlaI.1, R.LlaI.1 | Integrase, Transposase |
| *Lactococcus lactis pNP40* | 585 | 46487641 | AAS99179.1 | 46487642 | M1.LlaJI, M2.LlaJI, R2.LlaJI | Integrase |
| *Staphylococcus aureus subsp. aureus MRSA252* | 567 | 49482329 | YP_039553.1 | 49482330 | ND | Transposase x 3 |
| *Staphylococcus haemolyticus JCSC1435* | 498 | 70725087 | YP_252001.1 | 70725086 | ND | Integrase, Transposase |
| *Streptococcus mutans UA159* | 567 | 24379878 | NP_721833.1 | 24379877 | ND | ND |
| *Streptococcus pneumoniae D39* | 643 | 116515594 | YP_816579.1 | 116516577 | ND | ND |
| *Streptococcus pneumoniae R6* | 644 | 15903173 | NP_358723.1 | 15903172 | ND | ND |
| *Streptococcus pneumoniae SP11-BS70* | 643 | 147755520 | EDK62568.1 | 147755519 | ND | ND |
| *Streptococcus pneumoniae SP14-BS69* | 643 | 147759230 | EDK66223.1 | 147759229 | ND | ND |
| *Streptococcus pneumoniae SP18-BS74* | 643 | 147761390 | EDK68356.1 | 147761391 | ND | ND |
| *Streptococcus pneumoniae SP19-BS75* | 643 | 147763629 | EDK70564.1 | 147763628 | ND | ND |
| *Streptococcus pneumoniae SP23-BS72* | 307 | 147931113 | EDK82092.1 | 147931112 | ND | ND |
| *Streptococcus pneumoniae SP3-BS71* | 668 | 147922592 | EDK73710.1 | 147922591 | ND | ND |
| *Streptococcus pneumoniae SP6-BS73* | 643 | 147925252 | EDK76331.1 | 147925251 | ND | ND |
| *Streptococcus pneumoniae SP9-BS68* | 643 | 147927685 | EDK78710.1 | 147927684 | ND | ND |
| *Streptococcus pneumoniae TIGR4* | 643 | 111658426 | ZP_01409105.1 | 111658427 | ND | ND |
| *Streptococcus pyogenes MGAS10394* | 555 | 50914505 | YP_060477.1 | 50914504 | M1.Spy10394ORF1143P, M2.Spy10394ORF1143P M.Spy10394ORF1160P, Spy10394ORF1160P | Recombinase |
| *Streptococcus suis 05ZYH33* | 569 | 146318514 | YP_001198226.1 | 146318515 | ND | Phage integrase |
| *Streptococcus suis 98HAH33* | 223 | 146320708 | YP_001200419.1 | 146320709 | ND | Phage integrase |
| *Symbiobacterium thermophilum IAM 14863* | 609 | 51893430 | YP_076121.1 | 51893429 | ND | Transposase, *DDE*.Transposase, mutator, Integrase |
| *Syntrophomonas wolfei subsp. wolfei str. Goettingen* | 679 | 114566643 | YP_753797.1 | 114566644 | ND | ND |
| *Thermoanaerobacter ethanolicus ATCC 33223* | 803 | 76796475 | ZP_00778836.1 | 76796476 | ND | ND |
| *Thermoanaerobacter ethanolicus ATCC 33223* | 456 | 76795692 | ZP_00778064.1 | 76796891 remote, 76797610 remote | MTase | Integrase |
| *Thermoanaerobacter ethanolicus X514* | 619 | 114843463 | ZP_01453927.1 | 114843462 | ND | ND |
| *Thermoanaerobacter ethanolicus X514* | 583 | 114843808 | ZP_01454268.1 | 114843807 | Mrr, MTase, REase | ND |
|  |  |  |  |  |  |  |
| **Actinobacteria** |  |  |  |  |  |  |
| *Arthrobacter aurescens TC1* | 718 | 119962222 | YP_946857.1 | 119962877 remote | ND | ND |
| *Arthrobacter sp. FB24* | 743 | 116669427 | YP_830360.1 | 116669428 | ND | ND |
| *Corynebacterium glutamicum R* | 473 | 145294212 | YP_001137033.1 | 145294213 | ND | Integrase |
| *Frankia sp. CcI3* | 741 | 86741527 | YP_481927.1 | 86741526 | ND | ND |
| *Mycobacterium gilvum PYR-GCK* | 371 | 145225781 | YP_001136459.1 | 145225780, 145225779 frameshift, split gene | ND | ND |
| *Mycobacterium gilvum PYR-GCK* | 700 | 145223785 | YP_001134463.1 | 145223784 | ND | Transposase |
| *Mycobacterium sp. KMS* | 609 | 119866899 | YP_936851.1 | 119866898 | ND | ND |
| *Mycobacterium sp. MCS* | 609 | 108797806 | YP_638003.1 | 108797805 | V.MspMCSORF817P, M.MspMCSORF817P | Transposase (mutator) x 2, Transposase (IS*3*/IS*911*) |
| *Nocardioides sp. JS614* | 899 | 119714076 | YP_919218.1 | 119714075 | RM.NspJSORF4563P, M.NspJCORF4776P | ND |
| *Rhodococcus sp. RHA1* | 249 | 111020925  (degenerated) | YP_703897.1 | ND | ND | ND |
|  |  |  |  |  |  |  |
| **Bacteroidetes/chlorobi** |  |  |  |  |  |  |
| *Bacteroides fragilis NCTC 9343* | 575 | 60682387 | YP_212530.1 | 60682388 | MTase | ND |
| *Dokdonia donghaensis MED134* | 936 | 86130664 | ZP_01049264.1 | 86130665 | HindVII, HsdM, RmeS, HsdR | ND |
| *Flavobacteria bacterium BBFL7* | 989 | 89891074 | ZP_01202582.1 | 89891073 | HsdR, HsdM, HsdS | ND |
| *Flavobacterium psychrophilum JIP02/86* | 823 | 150024938 | YP_001295764.1 | 150024937 | ND | ND |
| *Microscilla marina ATCC 23134* | 433 | 124004104 | ZP_01688951.1 | 124008203 | ND | ND |
| *Pelodictyon luteolum DSM 273* | 606 | 78186706 | YP_374749.1 | 78186705 | ND | ND |
| *Porphyromonas gingivalis W83* | 571 | 34540720 | NP_905199.1 | 34540721 | PgiTMrrP | Transposase (IS*Pg4*) |
| *Psychroflexus torquis ATCC 700755* | 641 | 91216791 | ZP_01253755.1 | 91216792 | HsdM, HsdS, HsdR | ND |
| *Psychroflexus torquis ATCC 700755* | 514 | 91216809 | ZP_01253773.1 | 91216810 truncated | ND | ND |
|  |  |  |  |  |  |  |
| **Chloroflexi** |  |  |  |  |  |  |
| *Chloroflexus aggregans DSM 9485* | 542 | 118045679 | ZP_01514337.1 | 118047303 remote | ND | ND |
| *Chloroflexus aurantiacus J-10-fl* | 540 | 76260082 | ZP_00767723.1 | 76260687 remote | ND | ND |
| *Herpetosiphon aurantiacus ATCC 23779* | 549 | 113937422 | ZP_01423298.1 | 113938842 remote, 113938020 remote | ND | ND |
| *Roseiflexus castenholzii DSM 13941* | 377 | 156744006 | YP_001434135.1 | 118063015 remote | ND | ND |
| *Roseiflexus castenholzii DSM 13941* | 578 | 156744116 | YP_001434245.1 | ND | ND | ND |
| *Roseiflexus sp. RS-1* | 578 | 148654233 | YP_001274438.1 | 148656374 remote | ND | ND |
| *Roseiflexus sp. RS-1* | 378 | 148655039 | YP_001275244.1 | 148655179 remote | ND | ND |
| *Roseiflexus sp. RS-1* | 668 | 148655178 | YP_001275383.1 | ND | ND | ND |
|  |  |  |  |  |  |  |
| **Cyanobacteria** |  |  |  |  |  |  |
| *Crocosphaera watsonii WH 8501* | 506 | 67922732 | ZP_00516235.1 | 67922734, 67924573 remote | ND | ND |
| *Cyanothece sp. CCY 0110* | 540 | 126656651 | ZP_01727865.1 | 126656654 | ND | ND |
| *Lyngbya sp. PCC 8106* | 531 | 119490044 | ZP_01622668.1 | ND | ND | ND |
| *Lyngbya sp. PCC 8106* | 508 | 119486444 | ZP_01620502.1 | 119486443 | ND | ND |
| *Nodularia spumigena CCY 9414* | 363 | 119511119 | ZP_01630237.1 | 119511118 | MTase | ND |
| *Nostoc punctiforme PCC 73102* | 529 | 23126777 | ZP_00108663.1 | 23126776 | ND | ND |
| *Synechococcus sp. RS9917* | 123 | 87125171 | ZP_01081018.1 |  | MTase, REase | Transposase x 2 |
| *Synechocystis sp. PCC 6803* | 462 | 16329259 | NP_439987.1 | 16329258 | ND | ND |
| *Synechocystis sp. PCC 6803* | 730 | 38505837 | NP_942455.1 | ND | ND | Transposase, YhgA-like |
| *Trichodesmium erythraeum IMS101* | 539 | 113477076 | YP_723137.1 | 113477075 | ND | ND |
|  |  |  |  |  |  |  |
| **Deinococcus-Thermus** |  |  |  |  |  |  |
| *Deinococcus geothermalis DSM 11300* | 716 | 94985604 | YP_604968.1 | 94985603 | ND | ND |
| *Deinococcus radiodurans R1* | 678 | 15807928 | NP_285589.1 | ND | ND | Transposase |
| *Deinococcus radiodurans R1 plasmid MP1* | 969 | 10957435 | NP_051672.1 | 10957488 | ND | Transposase |
|  |  |  |  |  |  |  |
| **Acidobacteria** |  |  |  |  |  |  |
| *Acidobacteria bacterium Ellin345* | 805 | 94970780 | YP_592828.1 | 94970779 | M.Aba345ORF3758P, S.Aba345ORF3758P, R.Aba345ORF3758P | Transposase (IS*116*/IS*110*/IS*902* family) |
|  |  |  |  |  |  |  |
| **Aquificae** |  |  |  |  |  |  |
| *Aquifex aeolicus VF5* | 469 | 15606070 | NP_213447.1 | 15606069 | ND | ND |
|  |  |  |  |  |  |  |
| **Verrucomicrobia** |  |  |  |  |  |  |
| *Opitutaceae bacterium TAV2* | 577 | 151584602 | EDN48041.1 | 153889382 remote | MTase | Integrase, Transposase *DDE*, Recombinase |
|  |  |  |  |  |  |  |
| **Archaea** |  |  |  |  |  |  |
| **Crenarchaeota** |  |  |  |  |  |  |
| *Caldivirga maquilingensis IC-167* | 539 | 126353232 | ZP_01710243.1 | 126353234 | ND | ND |
| *Metallosphaera sedula DSM 5348* | 599 | 146303799 | YP_001191115.1 | 146303798 | ND | ND |
| *Pyrobaculum aerophilum str. IM2* | 359 | 18314246 | NP_560913.1 | 18314247 | ND | ND |
| *Pyrobaculum arsenaticum DSM 13514* | 523 | 145591281 | YP_001153283.1 | 145591280 | ND | ND |
| *Pyrobaculum calidifontis JCM 11548* | 524 | 126460463 | YP_001056741.1 | 126460462 | ND | ND |
| *Pyrobaculum islandicum DSM 4184* | 403 | 119873426 | YP_931433.1 | 119873425 | ND | ND |
| *Pyrobaculum islandicum DSM 4184* | 475 | 119871589 | YP_929596.1 | 119871590 | ND | ND |
| *Sulfolobus acidocaldarius DSM 639* | 582 | 70607714 | YP_256584.1 | 70607713 | ND | ND |
| *Staphylothermus marinus F1* | 540 | 126465477 | YP_001040586.1 | 126465476 | M.SmaF1ORF576P | ND |
| *Staphylothermus marinus F1* | 548 | 126465479 | YP_001040588.1 | 126465478 | M.SmaF1ORF576P | ND |
| *Sulfolobus solfataricus P2* | 559 | 15898169 | NP_342774.1 | 15898170 | ND | Transposase (IS*C1439*), Transposase (IS*C1316*) |
| *Sulfolobus tokodaii str. 7* | 551 | 15921409 | NP_377078.1 | 15921408 | ND | ND |
| *Sulfolobus tokodaii str. 7* | 683 | 15921411 | NP_377080.1 | 15921410 | ND | ND |
| **Euryarchaeota** |  |  |  |  |  |  |
| *Caldivirga maquilingensis IC-167* | 539 | 126353232 | ZP_01710243.1 | ND | ND | ND |
| *Haloarcula marismortui ATCC 43049* | 544 | 55379062 | YP_136912.1 | 55379063, 55379065 one protein interrupted by transposase | ND | Phage Integrase, Transposase (IS*H8*) |
| *Methanococcus maripaludis S2* | 604 | 45358318 | NP_987875.1 | 45358319 | M.MmaSORF735P | Integrase |
| *Methanosarcina acetivorans C2A* | 700 | 20090962 | NP_617037.1 | 20090961 | MacORF2116P, S.MacORF2116P, M.MacORF2116P, M.MacORF2098P, MacORF2103P | ND |
| *Methanosarcina barkeri str. Fusaro* | 705 | 73670717 | YP_306732.1 | 73670716 | MbaORF3269P, S.MbaORF3269P, M.MbaORF3269P | ND |
| *Methanospirillum hungatei JF-1* | 999 | 88602580 | YP_502758.1 | 88602581 | ND | ND |
| *Methanothermobacter thermautotrophicus str. Delta H* | 546 | 15678529 | NP_275644.1 | 15678530 | ND | ND |
| *Picrophilus torridus DSM 9790* | 523 | 48477100 | YP_022806.1 | 48477099 | ND | ND |
| *Pyrococcus horikoshii OT3* | 788 | 14590732 | NP_142802.1 | 14590731 | ND | ND |
| *Thermococcus kodakarensis KOD1* | 700 | 57640730 | YP_183208.1 | 57640729 | ND | Integrase |
| *Thermococcus kodakarensis KOD1* | 828 | 57640944 | YP_183422.1 | 57640945 | ND | ND |

Red: Restriction endonuclease homolog.

Green: Specificity subunit homolog.

Blue: Metyltransferase homolog.

Orange: Homology both with a restriction endonuclease and a methyltransferase.

Pink: Plasmid-encoded *mcrB* homolog.

Lavender: *mcrB* homolog apparently inserted into a restriction-modification gene complex.

^*^Not detected.

^†^MTase: Methyltransferase.

^‡^REase: Restriction endonuclease.

^§^Vsr: Very-short-patch-repair endonuclease.

^¶^remote: present elsewhere in the genome, but not in the immediate neighborhood of *mcrB*-like gene
