# Supplementary material for: Cell death upon epigenetic genome methylation: a novel function of methyl-specific deoxyribonucleases
Source: Genome Biol. 2008 Nov 21;9(11):R163. doi: 10.1186/gb-2008-9-11-r163 (PMC2614495; doi:10.1186/gb-2008-9-11-r163)
Supplement: Additional data file 2 — Phylogenetic trees of McrB and McrC. [file gb-2008-9-11-r163-S2.pdf]

Fig. S1

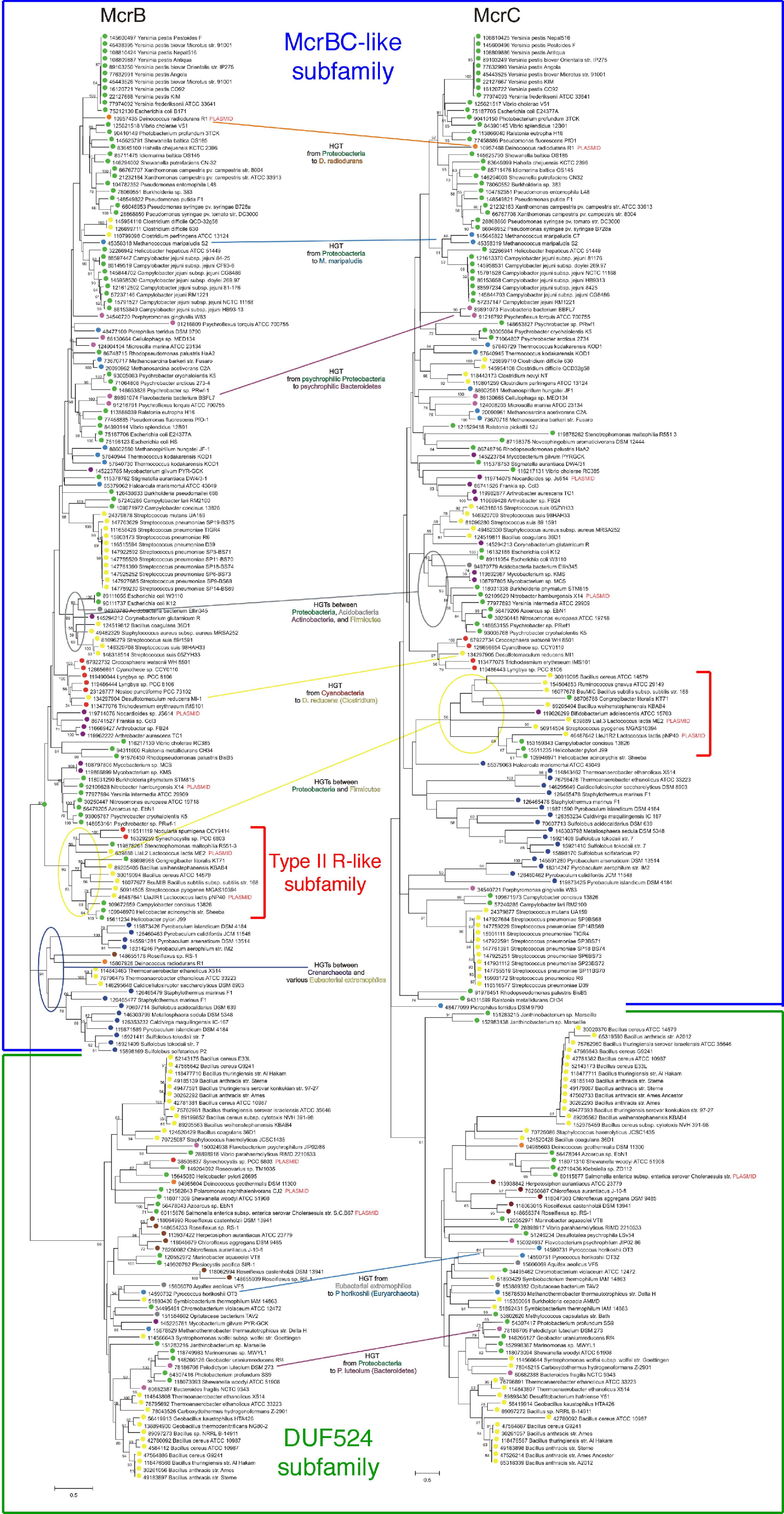
McrB
McrC
McrBC-like subfamily
HGT from Proteobacteria to D. radiodurans
HGT from Proteobacteria to M. maripaludis
HGT from psychrophilic Proteobacteria to psychrophilic Bacteroidetes
HGTs between Proteobacteria, Acidobacteria Actinobacteria, and Firmicutes
HGT from Cyanobacteria to D. reducens (Clostridium)
HGTs between Proteobacteria and Firmicutes
Type II R-like subfamily
HGTs between Crenarchaeota and various Eubacterial extremophiles
HGT from Eubacterial extremophiles to P horikoshii (Euryarchaeota)
HGT from Proteobacteria to P. luteolum (Bacteroidetes)
DUF524 subfamily
0.5
0.5

Figure S1 - Phylogenetic trees of McrB and McrC.

Minimum Evolution trees of McrB (left) and McrC (right) calculated with MEGA4 (see Materials and methods). Protein sequences are indicated by their NCBI Gene Identification number, followed by the genus and species name and the strain number. Membership in higher order taxons is indicated by color dots: green for Proteobacteria, yellow for Firmicutes, red for Cyanobacteria, violet for Bacteroidetes, brown for Chloroflexi, orange for the Deinococcus/Thermus group, light blue for Euryarchaeota, dark blue for Crenarchaeota, gray for others. Numbers at the nodes indicate bootstrap support in percent for particular bifurcations; only values > 50% are considered as reliable, while nodes without a number should be considered as unresolved. The potentially transferred McrB-McrC pairs have been indicated by lines colored according to the recipient/donor genomes.
