# Supplementary material for: Cell death upon epigenetic genome methylation: a novel function of methyl-specific deoxyribonucleases
Source: Genome Biol. 2008 Nov 21;9(11):R163. doi: 10.1186/gb-2008-9-11-r163 (PMC2614495; doi:10.1186/gb-2008-9-11-r163)
Supplement: Additional data file 3 — Neighbors of mcrB homologs. [file gb-2008-9-11-r163-S3.doc]

## Table S2. Neighbors of *mcrB* homologs.

|  | **McrBC neighborhood** | *E. coli* K12 genome | *B. subtilis* genome | *P. abyssi* genome | McrBC neighborhood | *E.coli* K12 genome | *B. subtilis* genome | *P. abyssi* genome | avg. of 3 genomes | McrBC/ *E. coli* | McrBC/ *B. subtilis* | McrBC/ *P. abyssi* | McrBC/ avg. 3 genomes |
| --- | --- | --- | --- | --- | --- | --- | --- | --- | --- | --- | --- | --- | --- |
| **Total number of proteins** | **2770** | **4031** | **3686** | **1700** | Fraction | | | | | **Ratio**  **(McrB neighb. vs genome)** | | | |
| **McrB** | 199 | 1 | 1 | 0 | 7.18% | 0.02% | 0.03% | 0.00% | 0.02% | 289.59 | 264.81 | N/A | **338.26** |
| **McrC** | 174 | 1 | 1 | 0 | 6.28% | 0.02% | 0.03% | 0.00% | 0.02% | 253.21 | 231.54 | N/A | **295.77** |
| **HSDS** | 21 | 1 | 0 | 1 | 0.76% | 0.02% | 0.00% | 0.06% | 0.02% | 30.56 | N/A | 12.89 | **35.70** |
| **DNA:m5C or m6A methyltransferase** | 21 | 3 | 4 | 3 | 0.76% | 0.07% | 0.11% | 0.18% | 0.11% | 10.19 | 6.99 | 4.30 | **7.14** |
| **integrase** | 59 | 33 | 7 | 1 | 2.13% | 0.82% | 0.19% | 0.06% | 0.44% | 2.60 | 11.22 | 36.21 | **4.89** |
| **transposase** | 57 | 53 | 9 | 4 | 2.06% | 1.31% | 0.24% | 0.24% | 0.70% | 1.57 | 8.43 | 8.75 | **2.94** |
| **HATPase** | 35 | 26 | 24 | 1 | 1.26% | 0.65% | 0.65% | 0.06% | 0.54% | 1.96 | 1.94 | 21.48 | **2.33** |
| **HTH_3** | 25 | 13 | 19 | 7 | 0.90% | 0.32% | 0.52% | 0.41% | 0.41% | 2.80 | 1.75 | 2.19 | **2.18** |
| **acetyltransferase** | 20 | 22 | 24 | 4 | 0.72% | 0.55% | 0.65% | 0.24% | 0.53% | 1.32 | 1.11 | 3.07 | **1.36** |
| **transporter** | 82 | 121 | 120 | 48 | 2.96% | 3.00% | 3.26% | 2.82% | 3.07% | 0.99 | 0.91 | 1.05 | **0.96** |
| **hydrolase** | 29 | 36 | 97 | 34 | 1.05% | 0.89% | 2.63% | 2.00% | 1.77% | 1.17 | 0.40 | 0.52 | **0.59** |
| **MFS_1** | 22 | 65 | 64 | 14 | 0.79% | 1.61% | 1.74% | 0.82% | 1.52% | 0.49 | 0.46 | 0.96 | **0.52** |

The relative frequency of members of different families is compared for McrB neighborhood and genomes of *E. coli* K12, *Bacillus subtilis*, and *Pyrococcus abyssi* (based on the HHSEARCH assignment, see Materials and methods). Homologs of McrC, DNA methyltransferases, specificity subunits of restriction enzymes, integrases, and transposases are those of the most frequent neighbors of McrB homologs, which are most overrepresented compared to the reference genomes.
