# Supplementary material for: Cell death upon epigenetic genome methylation: a novel function of methyl-specific deoxyribonucleases
Source: Genome Biol. 2008 Nov 21;9(11):R163. doi: 10.1186/gb-2008-9-11-r163 (PMC2614495; doi:10.1186/gb-2008-9-11-r163)
Supplement: Additional data file 4 — Phylogenetic tree of the 16S rRNA gene. [file gb-2008-9-11-r163-S4.pdf]

Fig. S2

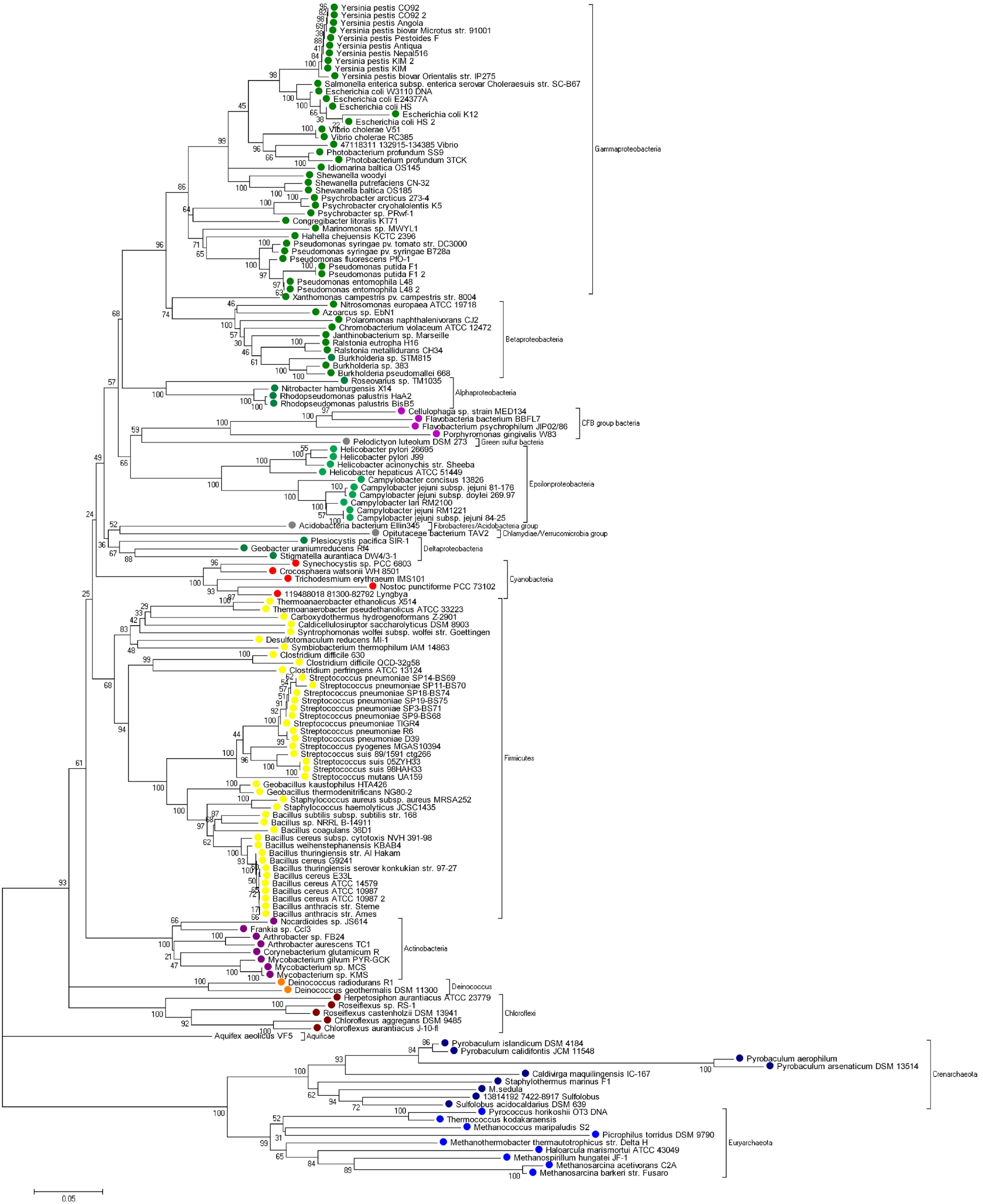
Yersinia pestis CO92
Yersinia pestis CO92 2
Yersinia pestis Angola
Yersinia pestis biovar Microtus str. 91001
Yersinia pestis Pestoides F
Yersinia pestis Antiqua
Yersinia pestis Nepal516
Yersinia pestis KIM 2
Yersinia pestis KIM
Yersinia pestis biovar Orientalis str. IP275
Salmonella enterica subsp. enterica serovar Choleraesuis str. SC-B67
Escherichia coli W3110 DNA
Escherichia coli E24377A
Escherichia coli HS
Escherichia coli K12
Escherichia coli HS 2
Vibrio cholerae V51
Vibrio cholerae RC385
47118311 132915-134385 Vibrio
Photobacterium profundum SS9
Photobacterium profundum 3TCK
Idiomarina baltica OS145
Shewanella woodyi
Shewanella putrefaciens CN-32
Shewanella baltica OS185
Psychrobacter arcticus 273-4
Psychrobacter cryohalolentis K5
Psychrobacter sp. PRwf-1
Congregibacter litoralis KT71
Marinomonas sp. MWYL1
Hahella chejuensis KCTC 2396
Pseudomonas syringae pv. tomato str. DC3000
Pseudomonas syringae pv. syringae B728a
Pseudomonas fluorescens PfO-1
Pseudomonas putida F1
Pseudomonas putida F1 2
Pseudomonas entomophila L48
Pseudomonas entomophila L48 2
Xanthomonas campestris pv. campestris str. 8004
Gammaproteobacteria
Nitrosomonas europaea ATCC 19718
Azoarcus sp. EbN1
Polaromonas naphthalenivorans CJ2
Chromobacterium violaceum ATCC 12472
Janthinobacterium sp. Marseille
Ralstonia eutropha H16
Ralstonia metallidurans CH34
Burkholderia sp. STM815
Burkholderia sp. 383
Burkholderia pseudomallei 668
Betaproteobacteria
Roseovarius sp. TM1035
Nitrobacter hamburgensis X14
Rhodopseudomonas palustris HaA2
Rhodopseudomonas palustris BisB5
Alphaproteobacteria
Cellulophaga sp. strain MED134
Flavobacteria bacterium BBFL7
Flavobacterium psychrophilum JIP02/86
Porphyromonas gingivalis W83
CFB group bacteria
Pelodictyon luteolum DSM 273
Green sulfur bacteria
Helicobacter pylori 26695
Helicobacter pylori J99
Helicobacter acinonychis str. Sheeba
Helicobacter hepaticus ATCC 51449
Campylobacter concisus 13826
Campylobacter jejuni subsp. jejuni 81-176
Campylobacter jejuni subsp. doylei 269.97
Campylobacter lari RM2100
Campylobacter jejuni RM1221
Campylobacter jejuni subsp. jejuni 84-25
Epsilonproteobacteria
Acidobacteria bacterium Ellin345
Fibrobacteres/Acidobacteria group
Opitutaceae bacterium TAV2
Chlamydiae/Verrucomicrobia group
Plesiocystis pacifica SIR-1
Geobacter uraniumreducens Rf4
Stigmatella aurantiaca DW4/3-1
Deltaproteobacteria
Synechocystis sp. PCC 6803
Crocosphaera watsonii WH 8501
Trichodesmium erythraeum IMS101
Nostoc punctiforme PCC 73102
119488018 81300-82792 Lyngbya
Cyanobacteria
Thermoanaerobacter ethanolicus X514
Thermoanaerobacter pseudethanolicus ATCC 33223
Carboxydothermus hydrogenoformans Z-2901
Caldicellulosiruptor saccharolyticus DSM 8903
Syntrophomonas wolfei subsp. wolfei str. Goettingen
Desulfotomaculum reducens MI-1
Symbiobacterium thermophilum IAM 14863
Clostridium difficile 630
Clostridium difficile QCD-32g58
Clostridium perfringens ATCC 13124
Streptococcus pneumoniae SP14-BS69
Streptococcus pneumoniae SP11-BS70
Streptococcus pneumoniae SP18-BS74
Streptococcus pneumoniae SP19-BS75
Streptococcus pneumoniae SP3-BS71
Streptococcus pneumoniae SP9-BS68
Streptococcus pneumoniae TIGR4
Streptococcus pneumoniae R6
Streptococcus pneumoniae D39
Streptococcus pyogenes MGAS10394
Streptococcus suis 89/1591 ctg266
Streptococcus suis 05ZYH33
Streptococcus suis 98HAH33
Streptococcus mutans UA159
Geobacillus kaustophilus HTA426
Geobacillus thermodenitrificans NG80-2
Staphylococcus aureus subsp. aureus MRSA252
Staphylococcus haemolyticus JCSC1435
Bacillus subtilis subsp. subtilis str. 168
Bacillus sp. NRRL B-14911
Bacillus coagulans 36D1
Bacillus cereus subsp. cytotoxis NVH 391-98
Bacillus weihenstephanensis KBAB4
Bacillus thuringiensis str. Al Hakam
Bacillus cereus G9241
Bacillus thuringiensis serovar konkukian str. 97-27
Bacillus cereus E33L
Bacillus cereus ATCC 14579
Bacillus cereus ATCC 10987
Bacillus cereus ATCC 10987 2
Bacillus anthracis str. Sterne
Bacillus anthracis str. Ames
Firmicutes
Nocardioides sp. JS614
Frankia sp. CcI3
Arthrobacter sp. FB24
Arthrobacter aurescens TC1
Corynebacterium glutamicum R
Mycobacterium gilvum PYR-GCK
Mycobacterium sp. MCS
Mycobacterium sp. KMS
Actinobacteria
Deinococcus radiodurans R1
Deinococcus geothermalis DSM 11300
Deinococcus
Herpetosiphon aurantiacus ATCC 23779
Roseiflexus sp. RS-1
Roseiflexus castenholzii DSM 13941
Chloroflexus aggregans DSM 9485
Chloroflexus aurantiacus J-10-fl
Chloroflexi
Aquifex aeolicus VF5
Aquificae
Pyrobaculum islandicum DSM 4184
Pyrobaculum calidifontis JCM 11548
Pyrobaculum aerophilum
Pyrobaculum arsenaticum DSM 13514
Caldivirga maquilingensis IC-167
Staphylothermus marinus F1
M.sedula
13814192 7422-8917 Sulfolobus
Sulfolobus acidocaldarius DSM 639
Crenarchaeota
Pyrococcus horikoshii OT3 DNA
Thermococcus kodakaraensis
Methanococcus maripaludis S2
Picrophilus torridus DSM 9790
Methanothermobacter thermautotrophicus str. Delta H
Haloarcula marismortui ATCC 43049
Methanospirillum hungatei JF-1
Methanosarcina acetivorans C2A
Methanosarcina barkeri str. Fusaro
Euryarchaeota
0.05

Figure S2 – A phylogenetic tree of 16S rRNA genes.

Minimum Evolution trees of 16S rRNA genes from the genomes carrying *mcrBC* homologs calculated with MEGA4 (see Materials and methods). The genus and species name and the strain number are indicated. Membership in higher order taxons is indicated by color dots: green for Proteobacteria, yellow for Firmicutes, red for Cyanobacteria, violet for Bacteroidetes, brown for Chloroflexi, orange for the Deinococcus/Thermus group, light blue for Euryarchaeota, dark blue for Crenarchaeota, gray for others. Numbers at the nodes indicate bootstrap support in percent for particular bifurcations; only values > 50% are considered as reliable, while nodes without a number should be considered as unresolved.
